# Fucosyl-Agalactosyl IgG_1_ Induces Cholangiocarcinoma Metastasis and Early Recurrence by Activating Tumor-Associated Macrophage

**DOI:** 10.3390/cancers10110460

**Published:** 2018-11-21

**Authors:** Ting-Tsung Chang, Hung-Wen Tsai, Cheng-Hsun Ho

**Affiliations:** 1 Department of Internal Medicine, National Cheng Kung University Hospital, College of Medicine, National Cheng Kung University, Tainan 70403, Taiwan; ttchang@mail.ncku.edu.tw; 2 Department of Pathology, National Cheng Kung University Hospital, College of Medicine, National Cheng Kung University, Tainan 70403, Taiwan; hungwen@mail.ncku.edu.tw; 3 Department of Medical Laboratory Science, College of Medicine, I-Shou University, Kaohsiung 82445, Taiwan

**Keywords:** cholangiocarcinoma, IgG, glycosylation, tumor-associated macrophage

## Abstract

Concern over roles of serum IgG agalactosylation in chronic inflammatory diseases has been mounting for years but less touched in cancers. The present study addressed the underlying role of agalactosylated IgG beyond tumorigenesis. Liquid-chromatography-tandem mass spectrometry was leveraged for the analysis of IgG_1_ and IgG_2_ *N*-glycomes. We found that a high percentage of serum fucosyl-agalactosyl IgG_1_ (IgG_1_-G0F) in patients with cholangiocarcinoma was associated with poor tumor differentiation and tumor metastasis. Results from Kaplan–Meier analyses and a stepwise Cox regression analysis showed that patients with serum IgG_1_-G0F ≥40% were highly correlated with poor recurrence-free survivals and overall survivals. Interestingly, patients with cholangiocarcinoma whose serum IgG_1_-G0F ≥40% had more CD163+ tumor-associated macrophages in cancerous tissues than adjacent non-cancerous counterparts. In vitro assays revealed that agalactosylated IgG upregulated tumor-associated macrophage markers CD163 and CD204 in human U-937 cells and peripheral macrophages. Moreover, a positive and a negative feedback loop of transforming growth factor-β1 and interferon-γ, respectively, on IgG agalactosylation was identified using hybridoma cells and verified in sera of the patients. In conclusion, agalactosylated IgG activates tumor-associated macrophages, thereby promoting tumor metastasis and recurrence of cholangiocarcinoma.

## 1. Introduction

Cholangiocarcinoma, emerging from a malignant proliferation of cholangiocytes, is an epithelial tumor of biliary trees. Primary sclerosing cholangitis, liver fluke infection (*Opisthorchis viverrini*), hepatolithiasis, biliary malformation (choledochal cysts or Caroli’s disease), and thorotrast are risk factors for the development of cholangiocarcinoma [1]. According to the location of tumor foci, cholangiocarcinoma is classified as intrahepatic type, a perihilar type (also known as Klatskin tumor), and a distal extrahepatic type [2]. Cholangiocarcinoma is the second most common (10 to 15%) primary hepatobiliary cancers after hepatocellular carcinoma and its incidence is approximately 2 per 100,000 population in western countries, nearly 5 per 100,000 population in Northeastern Asia, and even up to 85/100,000 in North Thailand [3,4,5]. Current diagnosis of cholangiocarcinoma mainly depends on ultrasonography. Surgical management is a curative treatment that is superior to chemotherapy, immunotherapy, and radiotherapy for cholangiocarcinoma. However, obscure clinical features and unsatisfactory sensitivity and specificity of ultrasonography drastically impede an early detection of this hidden killer in the early stage, resulting in high tumor metastatic and recurrent rates and eventually a poor prognosis in most patients [6]. The overall survival of patients with cholangiocarcinoma over the past 30 years is quite low, with less than 10% of cases survived for 5 years. Hence, it is an urgent need to identify biomarkers for early diagnosis and prognosis for cholangiocarcinoma [7].

Much more attentions on aberrant *N*-glycoproteome in chronic diseases and cancers have been drawn over the last decade owing to an advancement and application of high-throughput approaches, such as mass spectrometry, in clinical glycoresearches. For cholangiocarcinoma, investigations have been conducted using lectin-based determination of specific glycosylation patterns followed by glycoprotein identification in formalin-fixed, paraffin-embedded tissue specimens [8,9]. However, potential glycan and glycoprotein markers in circulation for the non-invasive diagnosis or prognosis of cholangiocarcinoma are less addressed. Immunoglobulin G (IgG), which bears *N*-linked glycans in the CH2 domain of the crystallizable fragment (Fc), is the most abundant *N*-glycoprotein in the circulation. The composition of glycan moiety on Fc has a profound effect on the Fcγ-receptor (FcγR) tropism of IgG and subsequent downstream immune responses. Agalactosylated IgG has been shown to elicit inflammation and mannan-binding lectin-mediated complement cascade [10] because of a high binding tendency to activating FcγRs [11]. In contrast, fully-galactosylated and sialylated IgG possesses a higher anti-inflammatory activity because it preferentially binds to the inhibitory FcγRIIB [12]. Based on our previous studies reporting a clinical relevance of aberrant serum IgG *N*-glycome in chronic hepatitis [13,14,15], we hypothesize a similar pathophysiological characteristic of IgG in bile duct diseases. Nevertheless, influences of IgG-Fc glycans on tumor progression remains an enigma. In this study, we report that agalactosylated IgG was able to activate tumor-associated macrophages, thereby leading to tumor metastasis and recurrence of cholangiocarcinoma.

## 2. Materials and Methods

### 2.1. Patients and Healthy Controls

This study was approved by the Institutional Review Board of National Cheng Kung University Hospital (NCKUH) (No. B-ER-103-133 and B-ER-105-098) and conducted in accord with the guidelines of the Declaration of Helsinki. Serum samples, laboratory data, tumor stage, and tumor differentiation grade of patients with intrahepatic cholangiocarcinoma (n = 50) and perihilar cholangiocarcinoma (n = 10) were obtained from the Tissue Bank, Research Center of Clinical Medicine, NCKUH. All the patients could not be identified. Serum samples and clinical data from healthy controls (n = 55) were obtained from the Health Examination Center of NCKUH. Participants who tested positive for human immunodeficiency virus, rheumatoid arthritis, juvenile onset chronic arthritis, systemic lupus erythematosus, or Crohn’s disease were excluded. Informed consent was obtained from each healthy donor. All samples were stored at −80 °C until use.

### 2.2. Enzyme-Linked Immunosorbent Assay (ELISA)

Total human IgG was detected using ELISA Quantitation Sets (Bethyl Laboratories, Montgomery, TX, USA). Interleukin (IL)-4, IL-6, IL-10, interferon (IFN)-γ, transforming growth factor (TGF)-β1, tumor necrosis factor (TNF)-α were detected using Ready-Set-Go ELISA kits (eBioscience, San Diego, CA, USA).

### 2.3. Purification of IgG and Generation of Agalactosyl IgG

Ten microliters of serum in 200 μL of phosphate-buffered saline (PBS) was incubated with 100 μL of Protein G-sepharose beads (GE Healthcare, Piscataway, NJ, USA) at room temperature for 2 h with gentle inversion. After 3 times of washings with 1× PBS, the beads were incubated with 0.1 M glycine-HCl (pH 2.8) at room temperature with vigorous vortexing to elute IgGs. Normal human serum IgG was purchased from Sigma-Aldrich (St. Louis, MO, USA). To generate sialic acids and galactose-free (asialyl-agalactosyl) IgG, normal serum IgG was treated with α2-3,6,8 neuraminidase and β1-4 galactosidase S (New England Biolabs, Ipswich, MA, USA) at 37 °C for 24 h. Normal serum IgG and asialyl-agalactosyl IgG were heated at 60 °C for 1 hour to generate aggregated IgG complexes.

### 2.4. Cell Culture and Treatment

A human monocytic cell line U-937 (Cat. NO. 60435) was purchased from Bioresource Collection and Research Center of Taiwan, which were originated from American Type Culture Collection. Human peripheral macrophages (Cat. No. 70042) were purchased from STEMCELL Technologies (Vancouver, Canada). A human cholangiocarcinoma cell line HuCCT1 (JCRB0425) and a highly differentiated, immortalized cholangiocyte cell line MMNK-1 (JCRB1554) were purchased from Japanese Collection of Research Bioresources Cell Bank. The generation of mouse hybridoma cells that produce mouse IgG_1_ has been previously described [16]. U-937 cells and human peripheral macrophages were cultured in Roswell Park Memorial Institute-1640 medium (Caisson Labs, Logan, UT, USA). HuCCT1 and MMNK-1 cells were cultured in Dulbecco’s Modified Eagle’s medium (Caisson Labs). Mouse hybridoma cells were cultured in Hybridoma serum-free medium (Thermo Fisher Scientific, Waltham, MA, USA). To avoid a non-specific crosslink of bovine IgGs in fetal bovine serum to human Fc gamma receptors [17], human cell lines were cultured in implicated media supplemented with 10% of heat-inactivated ultra-low IgG fetal bovine serum (Thermo Fisher Scientific), 100 U/mL of penicillin, and 100 μg/mL of streptomycin. All cells were cultured at 37 °C in the presence of 5% CO_2_ U-937 cells at the density of 5 × 10^5^/mL were treated with 10 ng/mL of phorbol 12-myristate 13-acetate (Sigma-Aldrich) for 48 h to induce macrophage differentiation. Hybridoma cells in fresh media at the density of 5 × 10^5^/mL were treated with different recombinant mouse cytokines (Cell Guidance Systems, Carlsbad, CA, USA) for 48 h.

### 2.5. Quantitative Reverse Transcription-Polymerase Chain Reaction (qRT-PCR)

Total RNAs were purified using REzol C&T (Protech Technology, Taipei, Taiwan). Five micrograms of total RNA was reverse transcribed using and Superscript III First-Strand Synthesis System (Thermo Fisher Scientific). PCR was performed using Power SYBR Green PCR Master Mix and StepOne Real-Time PCR System (Thermo Fisher Scientific). The PCR program was set in an initial step at 95 °C for 10 minutes with subsequently 40 cycles at 95 °C for 15 seconds and 56 °C for 1 minute. Sequences of primers for detecting tumor-associated macrophage markers are shown in Supplementary Table S1. The Ct value was determined using StepOne Software version 2.3 (Thermo Fisher Scientific).

### 2.6. Western Blot Analysis

Total cell lysates were resolved on sodium dodecyl sulfate-polyacrylamide gel and electrotransferred onto polyvinylidene fluoride membranes. After blocking in 5% (w/v) dry milk in Tris-buffered saline with 0.1% Tween 20, the membranes were incubated with anti-CD68 (ab125212, Abcam, Cambridge, UK), anti-CD163 (ab182422, Abcam), anti-CD204 (ab123946, Abcam), anti-CD64 (PA5-24855, Thermo Fisher Scientific), anti-CD16 (MA1-19006, Thermo Fisher Scientific), or anti-β-actin (ab8227, Abcam) primary antibody at room temperature for one hour. After washing, the membranes were incubated with horseradish peroxidase-conjugated goat anti-rabbit antibody (ab205718, Abcam). Signals were detected using a BioSpectrum Imaging System (UVP LLC, Upland, CA, USA).

### 2.7. Immunohistochemistry

The Bond–Max system (Leica Biosystems Newcastle Ltd., Newcastle, UK) was used for an automatically immunohistochemical staining. Formalin-fixed, paraffin-embedded tissue sections (4 µm thick) were de-paraffinized, rehydrated, and heated in 10 mM citrate buffer (pH 6.0) for antigen retrieval. Tissue sections were then incubated with mouse monoclonal antibody recognizing CD68 or CD163 (Leica Biosystems Newcastle Ltd.) at room temperature for 30 min. All slides were stained with 3,3’-diaminobenzidine substrate and counterstained with hematoxylin. Data were evaluated by a single experienced hepatopathologist who was blinded to clinical data of the patients.

### 2.8. Preparation of Tryptic Peptides

Membrane proteins from HuCCT1 and MMNK-1 cells were purified using Plasma Membrane Protein Extraction Kit (Abcam). IgGs or cell membrane proteins were denatured using 1% sodium dodecyl sulfate plus 10 mM dithiothreitol at 95 °C for 10 minutes and alkylated with 10 mM iodoacetamide at 37 °C in dark for 1 hour. Salt removal and protein concentration were conducted using Amicon Ultra-0.5 mL centrifugal filter (molecular weight cut-off 3000 daltons) device (Merck Millipore, Darmstadt, Germany). Proteins were quantified and digested with sequencing grade trypsin (Promega, Fitchburg, WI, USA) in an enzyme-to-substrate ratio of 1:50 at 37 °C overnight. Tryptic peptides were vacuum-dried and stored at −80 °C until analysis.

### 2.9. Liquid Chromatography–Tandem Mass Spectrometry (LC-MS/MS)

Samples were analyzed using Ultimate 3000 RSLC system (Dionex, Sunnyvale, CA, USA) coupled with a Q-Exactive mass spectrometer (Thermo Fisher Scientific). Mobile phase A was 0.1% fluoroacetic acid and mobile phase B was 0.1% fluoroacetic acid in 95% acetonitrile. The LC separation was performed using a C18 column (Acclaim PepMap RSLC, 75 μm × 150 mm, 2 μm, 100 Å) with the gradient consisting of (1) a linear increase from 1% to 25% B over 45 minutes, (2) a linear increase from 25% to 60% B over 10 minutes, and finally (3) an isocratic elution at 80% B for 10 minutes at 250 nL/minute for separation. A full MS scan was performed at the range of m/z 300–2000, and the ten most intense ions from MS scan were subjected to fragmentation for MS/MS analysis. Raw data was processed into peak lists by Proteome Discoverer 1.4 for Mascot database (2.4.0, Matrix Science Ltd., London, UK) search. For analyzing IgG glycan structure, selected ion chromatograms of different *N*-glycoforms attaching to the identified peptide backbones of human IgG_1_ heavy chain EEQYNSTYR and IgG_2_ heavy chain EEQFNSTFR were extracted from the raw data. Human IgG_3_ and IgG_4_ heavy chain peptide backbones share the same molecular weight that might not be distinguished by mass spectrometry and were therefore excluded in the *N*-glycomic analyses. The abundance of particular glycoforms on peptide backbones were estimated from the peak height or peak area divided by the sum of peak height/area of all major glycoforms extracted from the chromatogram acquired from the same run. MS^2^ spectra of each extracted glycopeptide were manually inspected to match all highly abundant product ions with a precursor ion mass accuracy <10 ppm to confirm their assignments. Other very low-abundant glycoforms on IgG_1_-Fc and IgG_2_-Fc, such as those with tri-antennary and tetra-antennary, were preliminarily excluded. Analysts were blinded to any information about the subjects.

### 2.10. Statistical Analysis

SPSS 17.0 for Windows was used for all statistical analyses. Continuous variables were compared using Mann–Whitney *U* tests for two independent groups and Kruskal–Wallis tests or one-way analysis of variance for three groups. Nominal variables were compared using Fisher’s exact tests or Pearson chi-square tests. The Pearson correlation coefficient (*r*) was used to evaluate the relationship between two groups. Receiver operator characteristic curves were used to evaluate IgG_1_-Fc and IgG_2_-Fc glycoforms on the differentiation of cholangiocarcinoma from healthy donors. Stepwise Cox regression analyses were performed to identify factors that were associated with early recurrence and overall survival of cholangiocarcinoma. Kaplan–Meier analyses and log-rank tests were used to assess the significance of IgG glycoforms on the recurrence-free survivals and post-surgical survivals of the patients with cholangiocarcinoma. Significance was defined as *p* < 0.05, and all *p*-values were two-tailed.

## 3. Results

### 3.1. Characteristics of Subjects

The demographic and clinical data of patients with cholangiocarcinoma, and sex and gender-matched healthy controls, are shown in Table 1. There was a 1:1 ratio of men to women. More than 40% of the patients were diagnosed with advanced-stage (≥3) tumors. Five-year recurrent rates in intrahepatic and perihilar cholangiocarcinomas were 40% and 50%, respectively. Five-year survival rates in two groups of patients were both lower than 20%. Patients had increased levels of alanine aminotransferase, aspartate aminotransferase, alkaline phosphatase, total bilirubin, glucose ante cibum, carcinoembryonic antigen, red cell distribution width, and white blood cell counts but had decreased levels of albumin, hemoglobin, and hematocrit. The patients had similar serum IgG level to the control subjects.

### 3.2. IgG-Fc N-Glycoprofiles in Cholangiocarcinoma

IgG_1_-Fc and IgG_2_-Fc *N*-glycosylation patterns in patients with cholangiocarcinoma and control subjects were analyzed using LC-MS/MS (Supplementary Table S2). Patients had higher proportions of FN, Man3FN, G0F, G0N, G0FN, G1N, and G2FN glycoforms in IgG_1_ and Man3FN, G0, G0F, and G1 glycoforms in IgG_2_ than did healthy controls (Table 2). Moreover, patients had decreased proportions of G1F and G2F glycoforms on IgG_1_ and G1F and G2FS glycoforms on IgG_2_. No differences in IgG_1_ and IgG_2_ glycosylation patterns between intrahepatic and perihilar cholangiocarcinomas were found (Supplementary Table S3). Moreover, results from receiver operator characteristic curves showed that the proportions of IgG_1_-G0F, IgG_1_-G0FN, IgG_1_-G1F, IgG_2_-G1, IgG_2_-G1F, and IgG_2_-G2FS possessed high sensitivity and specificity (greater than 70%) to differentiate the occurrence of cholangiocarcinoma (Table 3). Interestingly, only six glycoforms including G0FN, G1F, G1F2, G1FS, G2F, and G2FS between IgG_1_-Fc and IgG_2_-Fc were correlated (Supplementary Table S4). The distribution of the most abundant glycoform G0F on IgG_1_ and IgG_2_ in patients with cholangiocarcinoma was not correlated.

### 3.3. Association of IgG-Fc Glycans with Cholangiocarcinoma Differentiation, Metastasis, and Recurrence

**Figure 1:**
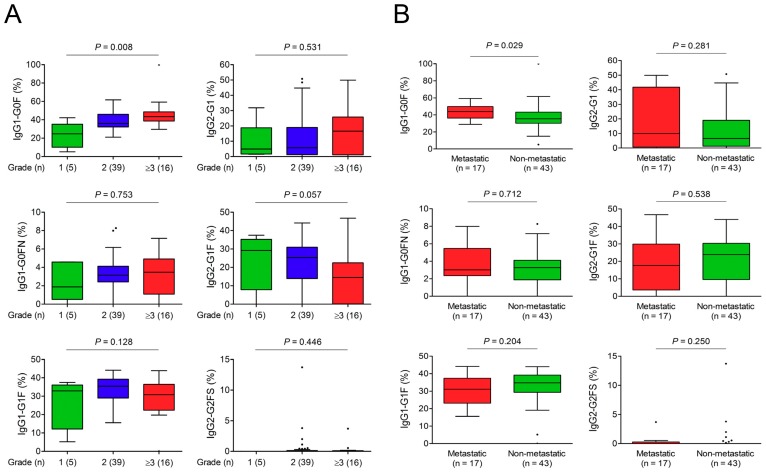

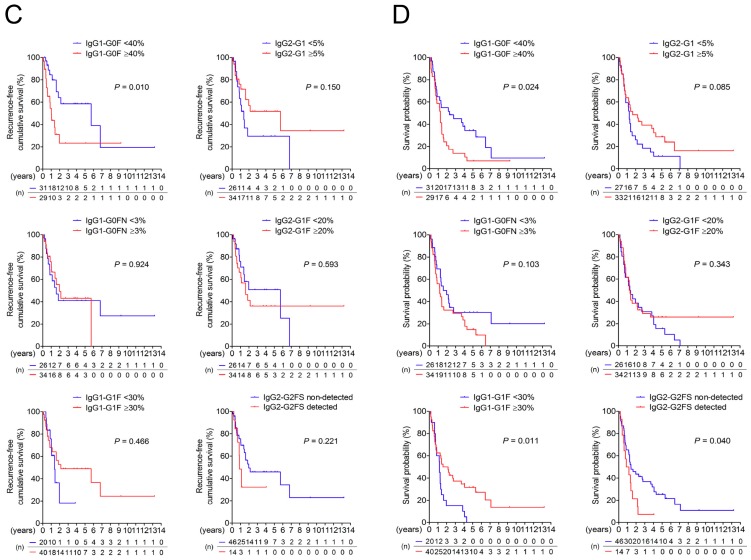
Association of IgG glycoforms and cholangiocarcinoma differentiation, metastasis, recurrence, and survivals. Levels of IgG_1_-G0F, IgG_1_-G0FN, IgG_1_-G1F, IgG_2_-G1, IgG_2_-G1F, and IgG_2_-G2FS (**A**) in different tumor grades and (**B**) with or without tumor metastasis are shown in Tukey box-and-whisker plots. *p*-values in (**A**) and (**B**) are obtained from Kruskal–Wallis tests and Mann–Whitney *U* tests, respectively. Kaplan–Meier analyses of (**C**) recurrence-free survivals and (**D**) overall survivals in patients with different levels of IgG_1_-G0F, IgG_1_-G0FN, IgG_1_-G1F, IgG_2_-G1, IgG_2_-G1F, and IgG_2_-G2FS are shown. *p*-values are obtained from log-rank tests. Abbreviations: F, fucosylated; G0, agalactosylated; G1, partially galactosylated; G2, fully galactosylated; N, bisected *N*-acetylglucosamine; S, sialylated.

There were no IgG glycoforms linking to the size of primary cholangiocarcinoma (Supplementary Table S5). Among six differential IgG glycoforms for cholangiocarcinoma, the proportion of IgG_1_-G0F was positively correlated with the tumor grade (Figure 1A). In addition, patients with lymph nodes or distal metastasis had an increased IgG_1_-G0F proportion (Figure 1B). Kaplan–Meier analyses using cut-off values around median levels revealed that IgG_1_-G0F was the only glycoform associated with the recurrence of cholangiocarcinoma (Figure 1C). Cox regression analyses indicated that IgG_1_-G0F ≥40% (hazard ratio 4.228 within 1 year and 3.505 within 2 years) was an independent factor for an early recurrence of cholangiocarcinoma (Table 4). Next, we studied IgG glycoforms that may affect a post-surgical survival in cholangiocarcinoma. Kaplan–Meier analyses revealed that patients with IgG_1_-G0F ≥40%, IgG_1_-G1F <30%, or detectable IgG_2_-G2FS had a lower post-surgical survival rate than the counterpart (Figure 1D). Results from Cox regression analyses showed that age was an important factor that was associated with a high 2- or 5-year mortality rate of cholangiocarcinoma after surgery (Supplementary Table S6). The significance of IgG glycoform in this model was dispensable when compared to other clinical factors.

### 3.4. Reciprocal Regulations of IgG-G0F and Tumor-Associated Macrophages

**Figure 2:**
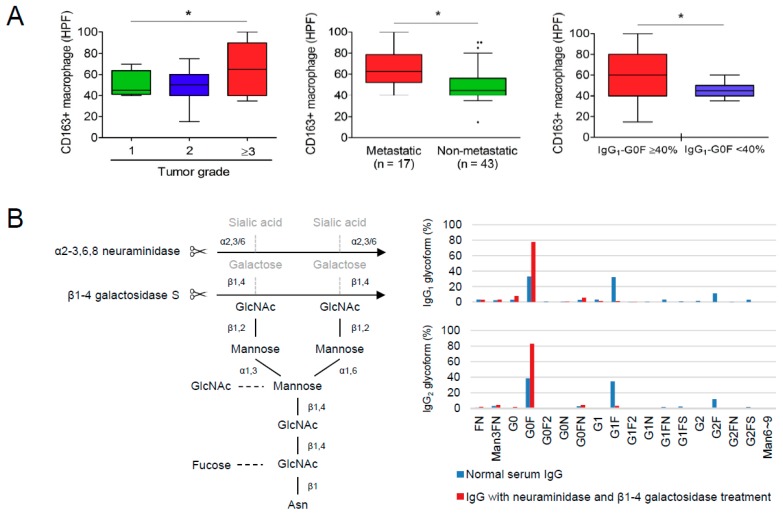

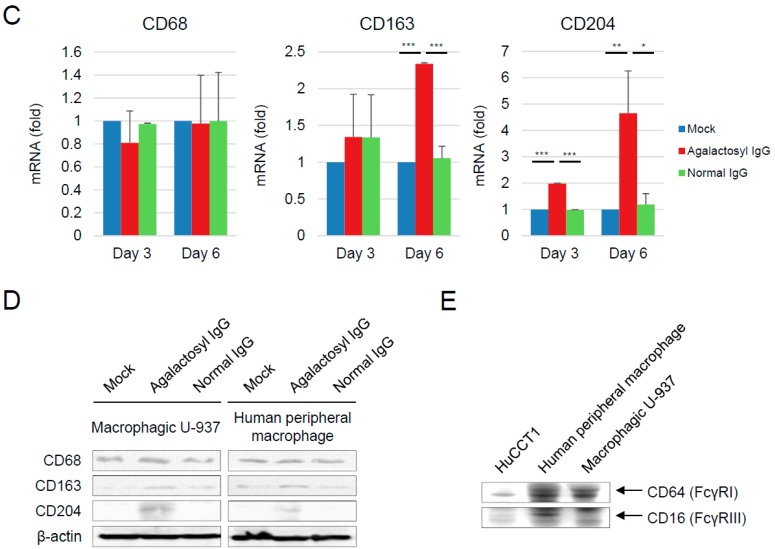
Induction of tumor-associated macrophage by agalactosylated IgG. (**A**) Numbers of CD163+ macrophages in the tumor foci of cholangiocarcinoma in patients with different tumor grades (left panel), with or without tumor metastasis (middle panel), or with different IgG_1_-G0F level (right panel) are shown in Tukey box-and-whisker plots. A *P*-value in the left panel is obtained from the Kruskal–Wallis test. *P*-values in the middle and right panels are obtained from Mann–Whitney *U* tests. (**B**) A schematic representation of the depletion of terminal sialic acid and galactose moieties on serum IgG using α2-3,6,8 neuraminidase and β1-4 galactosidase S, respectively. The proportion of each glycoform on normal or galactose-and-sialic acid-removed (asialyl-agalactosyl) IgG_1_ and IgG_2_ are shown. (**C**) Messenger RNA levels of the macrophage marker CD68 and tumor-associated macrophage markers CD163 and CD204 in U-937 cells after treatments with 10 ng/mL of phorbol 12-myristate 13-acetate for 2 days and 10 mg/mL of IgG (blue bar, mock; red bar, agalactosyl IgG; green bar, normal serum IgG) for another 3 or 6 days, are shown in bar graphs as means with standard deviations. Results are obtained from three independent experiments. *p*-values are obtained from one-way analysis of variance with Scheffé post hoc tests. Immunoblotting assays to detect protein levels of (**D**) CD68, CD163, and CD204 at day 6 post-treatment and (**E**) CD64 (FcγRI) and CD16 (FcγRIII) in macrophagic U-937 cells and human peripheral macrophages are shown. (* *p* < 0.05, ** *p* < 0.01, *** *p* < 0.001). Abbreviations: HPF, high-power field.

Because there were no Fc gamma receptors expressing on the surface of two cholangiocarcinoma cell lines HuCCT1 and MMNK-1 (Supplementary Table S7), a direct effect of IgG-G0F on the migration and invasion of cholangiocarcinoma cells was preliminarily excluded. Tumor-associated macrophages have been reported to be highly associated with a poorly-differentiated histology, tumor metastasis, and tumor recurrence of cholangiocarcinoma [18,19,20,21]. Immunohistochemistry results from 38 patients also showed that the level of CD163+ macrophage in cholangiocarcinoma tissues was associated with a poor differentiation of tumor cells and tumor metastasis (Figure 2A, left and middle panels). Moreover, a higher number of CD163+ macrophage in cancerous tissues was detected in the patients with serum IgG_1_-G0F ≥40% when compared to those with serum IgG_1_-G0F <40% (Figure 2A, right panel). Both IgG_1_-G0F and CD163+ macrophage in tumor foci were shown to link to an early cholangiocarcinoma recurrence in a univariate but not a multivariate Cox regression model (Table 4), suggesting that these two factors were tightly correlated. According to these clues, we assessed whether agalactosylated IgG connected to an activation of tumor-associated macrophages. We used commercial normal human serum IgG, of which IgG_1_ is the major component (60~70%), for the assays since we could not purify sufficient IgG_1_ proteins from human sera. Normal serum IgGs were treated with neuraminidase and β1-4 galactosidase to generate sialic acid and galactose-free (asialyl-agalactosyl) IgG (Figure 2B). The proportions of IgG_1_-G0F and IgG_2_-G0F in normal IgG reached about 80% in asialyl-agalactosyl IgG. Ten mg/mL of normal IgG or asialyl-agalactosyl IgG was used to treat macrophagic U-937 cells for a comparable IgG concentration in normal human serum. As we can see, the treatment of asialyl-agalactosyl IgG did not change an expression of a general macrophage marker CD68 but increased mRNA and protein levels of tumor-associated macrophage markers CD163 and CD204 in both macrophagic U-937 cells and human peripheral macrophages at day six (Figure 2C,D). Intriguingly, although agalactosylated IgGs have been reported to have a high priority to interact with FcγRIII [11], they might induce tumor-associated macrophages through a FcγRIII-independent manner because of the absence of FcγRIII expression in U-937 cells (Figure 2E).

**Figure 3:**
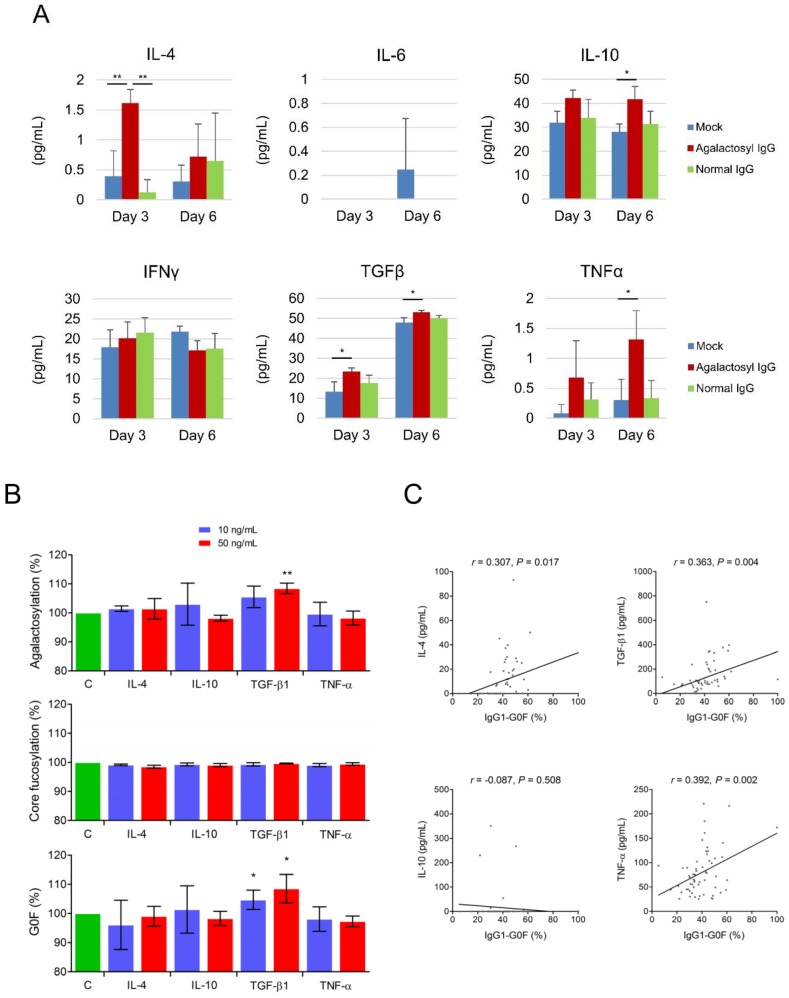
Regulation of IgG_1_-G0F. (**A**) Levels of tumor-associated macrophage-related cytokines in U-937 cells after treatments with 10 ng/mL of phorbol 12-myristate 13-acetate for 2 days and 10 mg/mL of IgG (blue bar, mock; red bar, agalactosyl IgG; green bar, normal serum IgG) for another 3 or 6 days, are shown in bar graphs as means with standard deviations. *P*-values are obtained from one-way analysis of variance with Scheffé post hoc tests. (**B**) Proportions of mouse IgG_1_ agalactosylation, core fucosylation, and G0F glycoform from mouse hybridoma cells after 48 h of cytokine treatment are shown in bar graphs as means with standard deviations. Results are obtained from three independent experiments. Two-tailed Student’s *t*-tests are used for comparisons between treatment groups (blue bar, 10 ng/mL; red bar, 50 ng/mL) and the control (C, green bar). *; *p* < 0.05; **; *p* < 0.01 (**C**) Correlations between IgG_1_-G0F and various cytokines in serum from the patients with cholangiocarcinoma (n = 60) are shown. The coefficient *r* is taken from Pearson’s correlation test. Abbreviations: IgG-G0F, agalactosylated and core fucosylated IgG; IFN, interferon; IL, interleukin; TGF, transforming growth factor; TNF, tumor necrosis factor.

Asialyl-agalactosyl IgG, as its effect on the activation of tumor-associated macrophages, upregulated tumor-associated macrophage-related cytokines including interleukin (IL)-4, IL-10, transforming growth factor (TGF)-β1, and tumor necrosis factor (TNF)-α, in macrophagic U-937 cells (Figure 3A). Next, we addressed whether these cytokines mutually regulate IgG agalactosylation. When using mouse IgG_1_-producing hybridoma cells in serum-free culture media as a model, we found that the treatment of recombinant mouse TGF-β1 stimulated IgG agalactosylation (Figure 3B). The trend of IgG_1_-G0F was similar to that of IgG agalactosylation because IgG_1_-G0F was the predominant fraction of the total agalactosylated IgG pool. None of these cytokines affected IgG core fucosylation. ELISA results revealed that levels of cytokines were all higher in the patients with cholangiocarcinoma than in the healthy controls (Supplementary Table S8). Moreover, the proportion of IgG_1_-G0F was positively correlated with levels of IL-4, TGF-β1, and TNF-α in sera of the patients (Figure 3C). These results indicated a vicious cycle between overactive agalactosylated IgG antibodies and tumor-associated macrophages.

## 4. Discussion

Aberrant serum total *N*-glycome is a prominently serological marker of chronic and end-stage liver diseases [22,23,24,25,26]. Being the most abundant circulating *N*-glycoprotein, IgG takes charge of the alternation in serum total *N*-glycome in disease status, even though it is not originated from the liver [27]. The immunomodulatory characteristic of IgG-Fc *N*-glycans [10,28,29,30] has a profound impact on the progression or treatment outcomes of liver disorders or cancers. Hence, serum IgG-Fc *N*-glycome may not be an ideally diagnostic marker but a potential prognostic marker in clinical practice. Pathologywise, cholangiocarcinoma impedes various biological functions of the liver and interferes with immune homeostasis. Accordingly, a manipulation and prognostic relevance of IgG-Fc *N*-glycans in biliary malignancies can be speculated. Here we report for the first time about the pathological role of agalactosylated IgG on the activation of tumor-associated macrophages, which links to tumor metastasis and early recurrence of cholangiocarcinoma.

Adults have the highest concentration of IgG_1_ (approximately 60–70%), followed by IgG_2_ (10–20%), IgG_3_ (4–8%), and IgG_4_ (2–5%). Four IgG subclasses possess distinct immune properties including Fc receptor tropism, complement cascade activation, and reaction to factors derived from other organisms [31,32]; moreover, they have different *N*-glycosylation patterns on the Fc portion [33]. There is more difference between healthy controls and cholangiocarcinoma patients for IgG_1_-Fc *N*-glycome than for IgG_2_-Fc *N*-glycome. Interestingly, even if glycan distributions between IgG_1_ and IgG_2_ were not synchronized, a unique glycoform G0F in both IgG subclasses were identified to be associated with the early recurrence of cholangiocarcinoma. Owing to a substantial limitation for dissecting IgG_3_-Fc (EEQYNSTFR) and IgG_4_-Fc (EEQFNSTYR) glycopeptides on mass spectra, we currently are not able to conclude the clinical significance of IgG_3_ and IgG_4_ glycosylation on the pathogenesis of the biliary duct system. Nevertheless, our findings make it practicable to apply IgG_1_ and IgG_2_ glycan analyses as routine laboratory tests for monitoring cholangiocarcinoma recurrence. A relatively weak prediction power of IgG-G0F on overall survivals may be influenced by extrinsic factors or medical interventions.

We have reported earlier that the proportion of IgG_1_-G0F was correlated with severities of liver necroinflammation and fibrosis, and it could also serve as a prognostic marker for a long-term antiviral therapy in patients with chronic hepatitis B [15]. As expected, the level of IgG-G0F was even higher in cholangiocarcinoma than in chronic hepatitis (39.6% versus 31.9%, *p* < 0.001) [14,15] owing to higher grades of liver deterioration and immune intricacy in hepatobiliary cancers. In regard to the associations of IgG-G0F with proinflammatory cytokines, tumor metastasis, and tumor recurrence, our results imply that agalactosylated IgG does not restrain but facilitate the expansion of biliary tumor cells, which stand for a theory of invalid inflammation on driving cancer cells proliferation and metastasis [34]. In vitro assays plus validations in tumor sections implicated agalactosylated IgG in the activation of tumor-associated macrophages. These notorious cells within tumor microenvironment are prone to promote tumor cell proliferation, angiogenesis, metastasis, matrix turnover, and a suppression of adaptive immunity [35,36]. Our findings, as well as other reports [19,20,21], demonstrate that a high density of tumor-associated macrophages in cancerous tissues is closely associated with a poor tumor cell differentiation, an extrahepatic metastasis, and a high recurrence rate in cholangiocarcinoma. Due to the steric hindrance of glycans (embed between two Fc domains) and a low immunogenicity of Fc glycopeptides, purification of intact IgG proteins bearing a specific glycoform using lectins or immunoprecipitation-based approaches currently remains a substantial challenge. Fortunately, agalactosyl IgG can be generated after the enzyme-based removal of terminal sialic acids and subsequent galactose moieties of the *N*-glycans on IgG. Because of the clinical relevance of IgG_1_-G0F in cholangiocarcinoma and its high abundance in the total agalactosylated IgG population, we focused on agalactosylated IgG to represent IgG_1_-G0F and excluded other IgG glycoforms, which have little or no relationships to the tumorigenesis of cholangiocarcinoma, in the assays of tumor-associated macrophages. An increase in the agalactosylated IgG during cholangiocarcinoma may lead to a drastic change in the binding preference to different FcγRs on the cell surface of macrophages. Though effector functions of galactosylated or biantennary α-2,6 sialylated IgG have been reported [37,38,39], the association between IgG_1_-G0F and tumor-associated macrophages may attribute to not only potent stimuli upon interactions between agalactosylated IgG-Fc and activating FcγRs, probably FcγRIII [11], but also a lack of blocking signals triggered by the binding of galactosyl/sialyl IgG to the inhibitory FcγRIIB [12]. Nonetheless, we currently cannot draw a conclusion on this issue since the unavailability of pure IgG_1_-G0F and the lack of FcγRIII expression in U-937 cells [40,41,42]. More analyses are needed to clarify whether other activating FcγRs or factors are involved in the IgG_1_-G0F-mediated induction of tumor-associated macrophages.

During the tumorigenesis of cholangiocarcinoma, cholestasis and chronic inflammation may induce bile duct epithelial cells to produce variable cytokines including IL-6, IL-8, TGF-β1, TNF-α, platelet-derived growth factor, and epidermal growth factor [43]. These mediators are known to promote the proliferation of bile duct epithelial cells and consequently contribute to the progression of cancer. The levels of these inflammatory mediators in the tumor microenvironment are shown to be higher than those in the circulation. Elevated levels of cytokines and other bioactive molecules, including IL-10, TGF-β1, and matrix metalloproteinase-2 have been demonstrated to be highly associated with M2 polarization of macrophages that surround cholangiocarcinoma tumor cells [20]. Moreover, tumor-associated macrophages are an important source of cytokines in the tumor microenvironment [44,45]. According to these clues and our findings, one may suspect that the network among IgG agalactosylation, cytokine, and tumor-associated macrophage might be stronger in tumor foci than what are observed from serum samples. Human peripheral CD19+ B cell is an ideal model to investigate the effect of cytokines on IgG glycosylation. However, primary B cells produce a very low amount of IgG without being activated. Priming stimuli, such as B cell receptor-triggering, CD40, IL-2, and IL-10, for activating peripheral B cells and complex factors in fetal bovine serum that were supplemented in culture medium make it difficult to individually dissect essential factor that may modulate IgG glycan biosynthesis. Therefore, we used mouse IgG_1_-producing hybridoma cells, which were cultured in Hybridoma serum-free medium, as a model since these cells generate a large number of IgG proteins in a stimulation-free mode, which let us get clear results about the effect of each cytokine on IgG glycan regulation with a minimum disturbance. We found that agalactosylated IgGs induced TGF-β1 overexpression, which facilitated the activation of tumor-associated macrophage. Unfortunately, an enhanced TGF-β1 secretions from tumor-associated macrophages may form a positive feedback loop on IgG agalactosylation, which may accelerate tumor progression. While the present study points out cholangiocarcinoma as the lead example, it is reasonable to speculate that the interplay between agalactosylated IgG and tumor-associated macrophage might be universal in other cancer types.

## 5. Conclusions

Agalactosylated IgG activates and forms a positive feedback loop with tumor-associated macrophages, thereby promoting tumor metastasis and early recurrence of cholangiocarcinoma.

## Figures and Tables

**Table 1:** Characteristics of healthy controls and patients with cholangiocarcinoma (CC).

Variable	Healthy Control (n = 55)	Intrahepatic CC (n = 50)	Perihilar CC (n = 10)	*p*-Value ^1^	*p*-Value ^2^	*p*-Value ^3^
**Demographic and cancer data**						
Male, n (%)	35 (63.6)	25 (50.0)	6 (60.0)	0.173	1.000	0.732
Age (years)	60 (56–80)	67 (33–85)	60 (38–77)	0.055	0.635	0.258
Tumor staging, n (%)						
I		10 (20)	1 (10)			
II		17 (34)	5 (50)			
III		4 (8)	2 (20)			
IVA		16 (32)	2 (20)			
IVB		3 (6)	0 (0)			
Follow-up period (years)		1.3 (0.2–13.2)	1.7 (0.1–5.5)			0.648
5-year recurrence, n (%)		20 (40)	5 (50)			0.728
5-year survivals, n (%)		8 (16)	2 (20)			0.668
**Biochemical tests**						
Alanine transaminase (U/L)	21 (9–45)	34 (10–199)	86 (13–140)	<0.001	<0.001	0.006
Aspartate aminotransferase (U/L)	23 (15–33)	42 (17–231)	62 (28–101)	<0.001	<0.001	0.031
Alkaline phosphatase (U/L)	64 (9–106)	118 (25–591)	246 (168–786)	<0.001	<0.001	0.001
Albumin (g/dL)	4.6 (4.2–5.2)	4.3 (2.9–4.9)	4.1 (3.0–5.2)	<0.001	<0.001	0.200
Immunoglobulin G (g/dL)	10.5 (4.3–23.5)	11.4 (8.3–15.8)	11.1 (8.7–13.0)	0.069	0.363	0.913
Total bilirubin (mg/dL)	0.9 (0.2–2.5)	0.5 (0.2–11.8)	5.0 (0.8–11.6)	0.002	<0.001	<0.001
Glucose ante cibum	87 (71–175)	103 (80–451)	109 (91–240)	<0.001	0.001	0.471
Creatinine (mg/dL)	0.93 (0.50–1.24)	0.80 (0.41–7.90)	0.80 (0.60–1.30)	<0.001	0.230	0.436
α-fetoprotein (ng/mL)	3.68 (1.82–16.83)	2.83 (1.31–474.20)	4.29 (1.76–5.79)	0.180	0.843	0.965
Carcinoembryonic antigen (ng/mL)	1.43 (0.44–4.09)	2.71 (0.30–60.42)	4.37 (1.53–13.18)	<0.001	<0.001	0.662
Carbohydrate antigen 125 (U/mL)		30.80 (7.95–772.10)	20.29 (9.15–201.80)			0.503
Carbohydrate antigen 19-9 (U/mL)		172.1 (0.0–36622.0)	330.9 (73.9–5791.0)			0.043
**Hematological tests**						
Red blood cell (10^6^/μL)	4.48 (3.56–5.90)	4.12 (3.17–5.47)	4.30 (2.56–5.57)	<0.001	0.068	0.796
Hemoglobin (g/dL)	14.1 (9.7–17.2)	12.8 (8.8–15.5)	12.4 (10.5–14.8)	<0.001	0.004	0.781
Hematocrit (%)	40.7 (29.1–49.2)	37.5 (25.6–44.7)	36.5 (31.0–42.7)	<0.001	0.004	0.677
Mean corpuscular hemoglobin (pg/cell)	31.5 (20.1–35.8)	31.1 (19.7–34.5)	31.8 (21.5–34.8)	0.045	0.806	0.425
Mean corpuscular hemoglobin concentration (g/dL)	34.4 (31.8–35.4)	33.9 (31.8–35.2)	34.2 (32.0–35.5)	<0.001	0.506	0.069
Mean corpuscular volume (fL/cell)	91.2 (63.1–101.5)	90.7 (61.9–101.0)	91.2 (67.1–102.5)	0.720	0.964	0.872
Red cell distribution width (%)	13.1 (11.8–21.8)	13.5 (12.0–17.7)	15.7 (11.9–25.9)	0.030	<0.001	0.004
White blood cell (10^3^/μL)	4.8 (3.1–7.8)	6.5 (3.6–16.9)	7.5 (4.2–12.1)	<0.001	<0.001	0.367
Platelet (10^3^/μL)	212 (70–317)	204 (84–412)	306 (149–394)	0.441	0.004	0.007

Data are numbers (percentages) or median values (minimum–maximum). Nominal values are compared using Fisher’s exact tests or Pearson chi-square tests. Continuous variables are compared using Mann–Whitney U tests. p-value ^1^, comparisons of values between healthy control and intrahepatic CC groups; p-value ^2^, comparisons of values between healthy control and perihilar CC groups; p-value ^3^, comparisons of values between intrahepatic CC and perihilar CC groups.

**Table 2:** IgG_1_-Fc and IgG_2_-Fc *N*-glycoprofiles in control subjects (n = 55) and in patients with cholangiocarcinoma (n = 60).

	IgG_1_			IgG_2_		
Glycoform	Control	Cholangiocarcinoma	*p*-Value	Control	Cholangiocarcinoma	*p*-Value
FN	0.00 (0.00–25.89)	0.16 (0.00–17.33)	<0.001	0.00 (0.00–13.17)	0.00 (0.00–44.26)	0.044
Man3FN	0.00 (0.00–0.31)	0.21 (0.00–6.42)	<0.001	0.00 (0.00–1.73)	0.00 (0.00–3.67)	<0.001
G0	0.00 (0.00–4.29)	1.97 (0.00–12.33)	<0.001	0.00 (0.00–3.15)	0.00 (0.00–4.05)	0.010
G0F	26.15 (0.00–52.62)	38.08 (5.20–100.00)	<0.001	32.55 (0.63–54.86)	39.68 (0.00–89.51)	0.012
G0F2	0.00 (0.00–0.00)	0.00 (0.00–8.00)	0.052	0.00 (0.00–34.72)	0.00 (0.00–15.23)	0.627
G0N	0.00 (0.00–1.03)	0.15 (0.00–1.28)	<0.001	0.00 (0.00–0.02)	0.00 (0.00–0.67)	0.020
G0FN	0.00 (0.00–7.05)	3.21 (0.00–8.27)	<0.001	1.55 (0.00–11.55)	2.58 (0.00–29.24)	0.023
G1	0.00 (0.00–7.50)	2.54 (0.00–9.18)	<0.001	0.00 (0.00–34.27)	6.86 (0.00–50.76)	<0.001
G1F	44.38 (8.59–100.00)	34.57 (0.00–44.14)	<0.001	37.02 (0.52–63.06)	22.81 (0.00–83.35)	<0.001
G1F2	0.00 (0.00–0.56)	0.00 (0.00–3.00)	0.359	0.00 (0.00–0.00)	0.00 (0.00–0.00)	1.000
G1N	0.00 (0.00–0.50)	0.12 (0.00–8.37)	<0.001	0.00 (0.00–1.40)	0.00 (0.00–14.53)	0.002
G1FN	0.00 (0.00–65.62)	2.47 (0.00–52.72)	0.008	0.00 (0.00–2.56)	0.25 (0.00–3.40)	0.006
G1FS	0.00 (0.00–0.61)	0.00 (0.00–25.99)	<0.001	1.74 (0.00–91.22)	0.09 (0.00–50.00)	<0.001
G2	0.00 (0.00–8.30)	0.89 (0.00–7.27)	0.002	0.00 (0.00–39.00)	1.28 (0.00–32.92)	0.035
G2F	19.15 (0.00–53.03)	10.83 (0.00–25.12)	<0.001	7.39 (0.00–35.23)	2.67 (0.00–21.80)	0.095
G2FN	0.00 (0.00–3.29)	0.16 (0.00–1.36)	<0.001	0.00 (0.00–0.14)	0.00 (0.00–0.77)	0.035
G2FS	0.00 (0.00–69.25)	0.00 (0.00–25.06)	0.010	1.39 (0.00–18.81)	0.00 (0.00–13.72)	<0.001
Man6~9	0.00 (0.00–0.00)	0.00 (0.00–79.28)	0.052	0.00 (0.00–26.48)	0.00 (0.00–20.41)	0.844

Data are median values (minimum–maximum). Mann–Whitney *U* tests were used to compare values between 2 groups. Abbreviations: F, fucosylated; G0, agalactosylated; G1, partially galactosylated; G2, fully galactosylated; Man, mannosylated; N, *N*-acetylglucosaminylated; S, sialylated.

**Table 3:** Differentiation of cholangiocarcinoma by IgG_1_-Fc and IgG_2_-Fc *N*-glycoforms.

	IgG_1_					IgG_2_				
Glycoform	AUC	SE	*p*-Value	Cut-off (%)	SENS, SPEC	AUC	SE	*p*-Value	Cut-off (%)	SENS, SPEC
FN	0.702	0.050	<0.001	0.02	58.3%, 85.5%	0.578	0.053	0.147		
Man3FN	0.813	0.041	<0.001	0.00	65.0%, 96.4%	0.653	0.051	0.005		
G0	0.777	0.044	<0.001	0.26	81.7%, 67.3%	0.606	0.053	0.050		
G0F	0.809	0.041	<0.001	30.28	81.7%, 70.9%	0.637	0.053	0.012		
G0F2	0.533	0.054	0.538			0.507	0.054	0.893		
G0N	0.762	0.046	<0.001	0.01	61.7%, 90.9%	0.559	0.054	0.279		
G0FN	0.795	0.043	<0.001	2.30	75.0%, 80.0%	0.621	0.052	0.025		
G1	0.684	0.051	<0.001			0.751	0.046	<0.001	1.12	78.3%, 70.9%
G1F	0.798 *	0.043	<0.001	38.26	72.7%, 78.3%	0.813 *	0.041	<0.001	30.49	80.0%, 76.7%
G1F2	0.516	0.054	0.771			0.500	0.054	1.000		
G1N	0.752	0.046	<0.001	0.03	56.7%, 92.7%	0.617	0.052	0.030		
G1FN	0.642	0.055	0.009			0.636	0.052	0.012		
G1FS	0.622	0.052	0.024			0.715 *	0.049	<0.001	1.24	61.8%, 76.7%
G2	0.662	0.053	0.003			0.608	0.054	0.046		
G2F	0.780 *	0.045	<0.001	16.36	61.8%, 85.0%	0.590	0.054	0.097		
G2FN	0.785	0.044	<0.001	0.11	61.7%, 96.4%	0.550	0.054	0.353		
G2FS	0.612	0.053	0.039			0.817 *	0.041	<0.001	0.03	80.0%, 76.7%
Man6~9	0.533	0.054	0.538			0.510	0.054	0.856		

* Decrease in cholangiocarcinoma. Abbreviations: AUC, area under receiver operating characteristic curve; F, fucosylated; G0, agalactosylated; G1, partially galactosylated; G2, fully galactosylated; Man, mannosylation; N, *N*-acetylglucosaminylated; S, sialylated; SE, standard error; SENS, sensitivity; SPEC, specificity.

**Table 4:** Cox regression analysis of early cholangiocarcinoma recurrence.

	Within 1 Year				Within 2 Years			
Variable	Hazard Ratio (95% CI)	*p*-Value	Hazard Ratio (95% CI)	*p*-Value	Hazard Ratio (95% CI)	*p*-Value	Hazard Ratio (95% CI)	*p*-Value
Sex (Male = 1. Female = 0)	0.520 (0.189–1.432)	0.206			0.668 (0.298–1.495)	0.326		
Age (years)	1.005 (0.965–1.047)	0.810			1.024 (0.990–1.059)	0.174		
Tumor type (Intrahepatic = 1, Perihilar = 0)	1.363 (0.309–6.007)	0.683			0.786 (0.292–2.113)	0.633		
Tumor stage	1.667 (0.829–3.352)	0.151			1.533 (0.886–2.651)	0.126		
Alanine transaminase (U/L)	1.000 (0.989–1.012)	0.984			1.005 (0.996–1.014)	0.305		
Aspartate aminotransferase (U/L)	0.999 (0.987–1.012)	0.922			1.001 (0.991–1.012)	0.836		
Alkaline phosphatase (U/L)	1.000 (0.995–1.005)	0.942			1.000 (0.995–1.004)	0.859		
Total bilirubin (mg/dL)	0.970 (0.795–1.185)	0.767			1.077 (0.958–1.211)	0.216		
α-fetoprotein (ng/mL)	0.964 (0.844–1.101)	0.588			0.992 (0.961–1.024)	0.604		
Carcinoembryonic antigen (ng/mL)	1.020 (0.990–1.050)	0.194			1.019 (0.990–1.050)	0.200		
Carbohydrate antigen 125 (U/mL)	1.001 (0.998–1.004)	0.590			1.000 (0.997–1.004)	0.771		
Carbohydrate antigen 19-9 (U/mL)	1.000 (1.000–1.000)	0.219			1.000 (0.997–1.004)	0.264		
Red blood cell (10^6^/μL)	1.001 (0.446–2.248)	0.998			0.879 (0.474–1.629)	0.681		
White blood cell (10^3^/μL)	0.998 (0.831–1.199)	0.986			0.998 (0.847–1.175)	0.980		
Platelet (10^3^/μL)	1.002 (0.995–1.008)	0.584			1.001 (0.996–1.006)	0.692		
IgG_1_-G0F ≥40% (Yes =1. No = 0)	4.228 (1.361–13.133)	0.013	2.765 (0.543–14.092)	0.221	3.505 (1.484–8.278)	0.004	2.521 (0.734–8.662)	0.142
CD68+ macrophage in tumor foci	1.023 (0.996–1.051)	0.098			1.019 (0.993–1.045)	0.156		
CD163+ macrophages in tumor foci	1.031 (1.004–1.059)	0.024	1.022 (0.994–1.051)	0.124	1.026 (1.002–1.050)	0.036	1.016 (0.991–1.041)	0.223

Abbreviations: CI, confidence interval; F, fucosylated; G0, agalactosylated.

## Supplementary Materials

The following are available online at http://www.mdpi.com/2072-6694/10/11/460/s1, Table S1: Primers for quantitative reverse transcription polymerase chain reaction; Table S2: Mass spectrometry-based analysis of human IgG1 and IgG2 N-glycosylation; Table S3: IgG1-Fc and IgG2-Fc N-glycoprofiles in patients with intrahepatic CC (n = 50) or perihilar CC (n = 10); Table S4: Relation between IgG1 and IgG2 glycoforms in patients with cholangiocarcinoma (n = 60); Table S5: Relation between IgG glycoforms and the tumor size of primary cholangiocarcinoma; Table S6: Cox regression analysis of mortality in cholangiocarcinoma; Table S7: Membrane protein identification in cholangiocarcinoma cell lines by mass spectrometry; Table S8: Levels of tumor-associated cytokines in healthy controls (n = 55) and patients with cholangiocarcinoma (n = 60).

## Abbreviations

CI	confidence interval
dHex	deoxyhexose
ELISA	enzyme-linked immunosorbent assay
F for glycoform	fucosylated
Fc	crystallizable fragment
FcγR	gamma receptor for crystallizable fragment
G0 for glycoform	agalactosylated
G1 for glycoform	partially galactosylated
G2 for glycoform	fully galactosylated
GlcNAc or N for glycoform	*N*-acetylglucosamine
Hex	hexose
HexNAc	*N*-acetylhexoseamine
IFN	interferon
IgG	immunoglobulin G
IL	interleukin
LC-MS/MS	Liquid chromatography-tandem mass spectrometry
Man	mannosylated
ROC curve	Receiver operating characteristic curve
S for glycoform	sialylated
TGF	transforming growth factor
TNF	tumor necrosis factor
