# Supplementary material for: Fucosyl-Agalactosyl IgG1 Induces Cholangiocarcinoma Metastasis and Early Recurrence by Activating Tumor-Associated Macrophage
Source: Cancers (Basel). 2018 Nov 21;10(11):460. doi: 10.3390/cancers10110460 (PMC6267046; doi:10.3390/cancers10110460)
Supplement: Supplementary file 1 [file cancers-10-00460-s001.pdf]

*Supplementary material*

# Fucosyl-Agalactosyl IgG1 Induces Cholangiocarcinoma Metastasis and Early Recurrence by Activating Tumor-Associated Macrophage

**Ting-Tsung Chang, Hung-Wen Tsai and Cheng-Hsun Ho**

**Table S1.** Primers for quantitative reverse transcription polymerase chain reaction.

| | Forward | Reverse |
|---|---|---|
| CD68 | 5′-CTTTGGGCAAAGTTTCTCCTGCC | 5′-CTCCGGATGATGCAGAAAGC |
| CD163 | 5′-AGCATGGAAGCGGTCTCTGTGATT | 5′-AGCTGACTCATTCCCACGACAAGA |
| CD204 | 5′-CTCCCCTTTTCCCCTTTCTG | 5'-ATCGAGGTCCCACTGGAGAAAGT |
| β-actin | 5'-CACCATTGGCAATGAGCGGTTC | 5'-AGGTCTTTGCGGATGTCCACGT |

CD68, macrophage marker; CD163 and CD204, tumor-associated macrophage markers.

**Table S2.** Mass spectrometry-based analysis of human IgG1 and IgG2 *N*-glycosylation.

| Glycan | Deduced Glycoform | Glyco-Structure | m/z of IgG1-Fc Glycopeptide EEQYNSTYR | m/z of IgG2-Fc Glycopeptide EEQFNSTFR |
|---|---|---|---|---|
| HexNAc1dHex1 | FN |  | 769.83 (2+) | 753.84 (2+) |
| Hex3HexNAc3dHex1 | Man3FN | 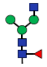 | 1215.98 (2+) | 1200.00 (2+) |
| Hex3HexNAc4 | G0 | 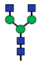 | 1244.50 (2+) | 1228.51 (2+) |
| Hex3HexNAc4dHex1 | G0F | 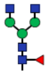 | 1317.53 (2+) | 1301.54 (2+) |
| Hex3HexNAc4dHex2 | G0F2 | 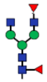 | 1390.56 (2+) | 1374.57 (2+) |
| Hex3HexNAc5 | G0N | 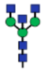 | 1346.04 (2+) | 1330.05 (2+) |
| Hex3HexNAc5dHex1 | G0FN | 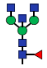 | 1419.07 (2+) | 1403.07 (2+) |

| | | | | |
|---|---|---|---|---|
| Hex4HexNAc4 | G1 | 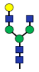 | 1325.52 (2+) | 1309.53 (2+) |
| Hex4HexNAc4dHex1 | G1F | 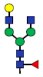 | 1398.55 (2+) | 1382.56 (2+) |
| Hex4HexNAc4dHex2 | G1F2 | 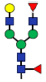 | 1471.58 (2+) | 1455.59 (2+) |
| Hex4HexNAc5 | G1N | 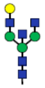 | 1427.06 (2+) | 1411.07 (2+) |
| Hex4HexNAc5dHex1 | G1FN | 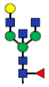 | 1500.09 (2+) | 1484.10 (2+) |
| Hex4HexNAc4dHex1NeuAc1 | G1FS | 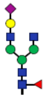 | 1544.10 (2+) | 1528.11 (2+) |
| Hex5HexNAc4 | G2 | 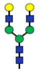 | 1406.55 (2+) | 1390.56 (2+) |
| Hex5HexNAc4dHex1 | G2F | 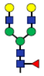 | 1479.58 (2+) | 1463.59 (2+) |
| Hex5HexNAc5dHex1 | G2FN | 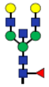 | 1581.12 (2+) | 1565.13 (2+) |
| Hex5HexNAc4dHex1NeuAc1 | G2FS | 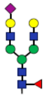 | 1625.14 (2+) | 1609.15 (2+) |
| Hex6~9HexNAc3 | Man6~9 | 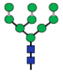 | | |

Abbreviations: dHex, deoxyhexose; F or Red triangle, fucose; G0, agalactosylated; G1 or single yellow circle, partially galactosylated; G2 or double yellow circles, fully galactosylated; Hex, hexose; HexNAc, *N*-acetylhexoseamine; Man or green circle, mannose; N or blue square, *N*-acetylglucosame; NeuAc, *N*-acetylneuraminic acid; S or purple diamond, sialylated.

**Table S3.** $IgG_1$-Fc and $IgG_2$-Fc *N*-glycoprofiles in patients with intrahepatic CC (n = 50) or perihilar CC (n = 10).

| | $IgG_1$ | | | $IgG_2$ | | |
|---|---|---|---|---|---|---|
| **Glycoform** | **Intrahepatic CC** | **Perihilar CC** | ***p*-Value** | **Intrahepatic CC** | **Perihilar CC** | ***p*-Value** |
| FN | 0.18 (0.00–4.24) | 0.02 (0.00–17.33) | 0.497 | 0.00 (0.00–20.64) | 0.00 (0.00–44.26) | 0.893 |
| Man3FN | 0.24 (0.00–6.42) | 0.00 (0.00–0.89) | 0.224 | 0.00 (0.00–2.11) | 0.00 (0.00–3.67) | 0.845 |
| G0 | 2.26 (0.00–12.33) | 0.90 (0.00–7.63) | 0.061 | 0.00 (0.00–4.05) | 0.00 (0.00–2.79) | 0.889 |
| G0F | 37.68 (5.20–61.72) | 41.82 (23.62–100.00) | 0.463 | 39.75 (0.00–89.51) | 39.01 (0.00–61.56) | 0.736 |
| G0F2 | 0.00 (0.00–8.00) | 0.00 (0.00–0.00) | 0.359 | 0.00 (0.00–15.23) | 0.00 (0.00–0.00) | 0.524 |
| G0N | 0.23 (0.00–1.28) | 0.00 (0.00–0.77) | 0.060 | 0.00 (0.00–0.67) | 0.00 (0.00–0.02) | 0.650 |
| G0FN | 3.18 (0.00–8.27) | 3.31 (0.00–6.17) | 0.781 | 2.43 (0.00–29.24) | 2.97 (0.00–4.57) | 0.889 |
| G1 | 2.71 (0.00–9.18) | 1.05 (0.00–4.89) | 0.024 | 7.23 (0.00–50.76) | 3.86 (0.00–44.34) | 0.292 |
| G1F | 33.78 (5.20–44.14) | 34.71 (0.00–42.84) | 0.921 | 22.30 (0.00–83.35) | 23.56 (0.00–46.78) | 0.538 |
| G1F2 | 0.00 (0.00–3.00) | 0.00 (0.00–0.00) | 0.431 | 0.00 (0.00–0.00) | 0.00 (0.00–0.00) | 1.000 |
| G1N | 0.13 (0.00–8.37) | 0.05 (0.00–2.00) | 0.583 | 0.00 (0.00–6.81) | 0.00 (0.00–14.53) | 0.874 |
| G1FN | 2.55 (0.00–52.72) | 1.02 (0.00–4.96) | 0.136 | 0.20 (0.00–3.40) | 0.33 (0.00–3.14) | 0.628 |
| G1FS | 0.00 (0.00–8.23) | 0.00 (0.00–25.99) | 0.279 | 0.05 (0.00–8.24) | 1.15 (0.00–50.00) | 0.092 |
| G2 | 1.00 (0.00–2.92) | 0.21 (0.00–7.27) | 0.236 | 1.03 (0.00–32.92) | 2.59 (0.00–12.27) | 0.785 |
| G2F | 11.32 (0.00–25.12) | 10.32 (0.00–16.18) | 0.346 | 3.03 (0.00–21.80) | 1.11 (0.00–14.69) | 0.347 |
| G2FN | 0.16 (0.00–1.36) | 0.18 (0.00–0.77) | 0.458 | 0.00 (0.00–0.48) | 0.00 (0.00–0.77) | 0.355 |
| G2FS | 0.00 (0.00–25.06) | 0.00 (0.00–13.72) | 0.172 | 0.00 (0.00–3.79) | 0.00 (0.00–13.72) | 0.370 |
| Man6~9 | 0.00 (0.00–79.28) | 0.00 (0.00–0.00) | 0.359 | 0.07 (0.00–20.41) | 0.00 (0.00–7.84) | 0.370 |

Data are median values (minimum–maximum). Mann–Whitney *U* tests were used to compare values between 2 groups. Abbreviations: CC, cholangiocarcinoma; F, fucosylated; G0, agalactosylated; G1, partially galactosylated; G2, fully galactosylated; Man, mannosylated; N, *N*-acetylglucosaminylated; S, sialylated.

**Table S4.** Relation between $IgG_1$ and $IgG_2$ glycoforms in patients with cholangiocarcinoma (n = 60).

| Glycoform | Coefficient *r* | *p*-Value |
|---|---|---|
| FN | −0.040 | 0.759 |
| Man3FN | −0.091 | 0.489 |
| G0 | 0.189 | 0.148 |
| G0F | −0.095 | 0.472 |
| G0F2 | −0.014 | 0.914 |
| G0N | 0.192 | 0.141 |
| G0FN | 0.432 | <0.001 |
| G1 | 0.022 | 0.870 |
| G1F | 0.419 | <0.001 |
| G1F2 | 0.988 | <0.001 |
| G1N | −0.037 | 0.779 |
| G1FN | −0.015 | 0.907 |
| G1FS | 0.404 | 0.001 |
| G2 | 0.195 | 0.135 |
| G2F | 0.538 | <0.001 |
| G2FN | 0.162 | 0.216 |
| G2FS | 0.403 | 0.001 |
| Man6~9 | −0.088 | 0.506 |

Results are obtained from Pearson correlation tests. Abbreviations: F, fucosylated; G0, agalactosylated; G1, partially galactosylated; G2, fully galactosylated; Man, mannosylated; N, *N*-acetylglucosaminylated; S, sialylated.

**Table S5.** Relation between IgG glycoforms and the tumor size of primary cholangiocarcinoma.

| Glycoform | $IgG_1$ *p*-Value | $IgG_2$ *p*-Value |
|---|---|---|
| FN | 0.630 | 0.426 |
| Man3FN | 0.876 | 0.654 |
| G0 | 0.439 | 0.861 |
| G0F | 0.319 | 0.456 |
| G0F2 | 0.377 | 0.831 |
| G0N | 0.691 | 0.807 |
| G0FN | 0.500 | 0.444 |
| G1 | 0.241 | 0.713 |
| G1F | 0.285 | 0.788 |
| G1F2 | 0.771 | 0.786 |
| G1N | 0.723 | 0.161 |
| G1FN | 0.966 | 0.729 |
| G1FS | 0.278 | 0.197 |
| G2 | 0.421 | 0.405 |
| G2F | 0.183 | 0.625 |
| G2FN | 0.489 | 0.298 |
| G2FS | 0.108 | 0.381 |
| Man6~9 | 0.331 | 0.202 |

*p*-values are obtained from Kruskal-Wallis tests. Abbreviations: F, fucosylated; G0, agalactosylated; G1, partially galactosylated; G2, fully galactosylated; Man, mannosylated; N, *N*-acetylglucosaminylated; S, sialylated.

**Table S6.** Cox regression analysis of mortality in cholangiocarcinoma.

| | Within 2 Years | | | | Within 5 Years | | | |
|---|---|---|---|---|---|---|---|---|
| | Univariate | | Multivariate | | Univariate | | Multivariate | |
| **Variable** | **Hazard Ratio (95% CI)** | ***p*-value** | **Hazard Ratio (95% CI)** | ***p*-Value** | **Hazard Ratio (95% CI)** | ***p*-value** | **Hazard Ratio (95% CI)** | ***p*-Value** |
| Sex (Male = 1. Female = 0) | 0.684 (0.356–1.314) | 0.254 | | | 0.768 (0.439–1.342) | 0.353 | | |
| Age (years) | 1.050 (1.019–1.083) | 0.002 | 1.043 (1.004–1.082) | 0.029 | 1.043 (1.017–1.070) | 0.001 | 1.046 (1.010–1.084) | 0.012 |
| Tumor type (Intrahepatic = 1, Perihilar = 0) | 1.175 (0.489–2.821) | 0.718 | | | 1.162 (0.545–2.478) | 0.698 | | |
| Tumor stage | 2.336 (1.406–3.881) | 0.001 | 1.628 (0.891–2.975) | 0.113 | 1.908 (1.268–2.871) | 0.002 | 1.541 (0.768–3.091) | 0.224 |
| Tumor grade | 1.438 (0.818–2.529) | 0.207 | | | 1.405 (0.862–2.289) | 0.172 | | |
| Alanine transaminase (U/L) | 0.999 (0.991–1.007) | 0.781 | | | 0.998 (0.991–1.005) | 0.535 | | |
| Aspartate aminotransferase (U/L) | 1.004 (0.996–1.011) | 0.343 | | | 1.003 (0.996–1.010) | 0.413 | | |
| Alkaline phosphatase (U/L) | 1.003 (1.001–1.006) | 0.004 | 1.003 (1.000–1.005) | 0.075 | 1.004 (1.001–1.006) | 0.001 | 1.004 (1.000–1.007) | 0.030 |
| Total bilirubin (mg/dL) | 1.025 (0.926–1.136) | 0.631 | | | 0.994 (0.904–1.092) | 0.893 | | |
| $\alpha$-fetoprotein (ng/mL) | 0.968 (0.886–1.057) | 0.463 | | | 0.964 (0.895–1.039) | 0.334 | | |
| Carcinoembryonic antigen (ng/mL) | 1.020 (0.999–1.041) | 0.057 | | | 1.022 (1.002–1.042) | 0.030 | 1.044 (1.010–1.079) | 0.012 |
| Carbohydrate antigen 125 (U/mL) | 1.001 (0.998–1.003) | 0.682 | | | 1.001 (0.999–1.003) | 0.517 | | |
| Carbohydrate antigen 19-9 (U/mL) | 1.000 (1.000–1.000) | 0.634 | | | 1.000 (1.000–1.000) | 0.545 | | |
| Red blood cell ($10^6$/μL) | 0.711 (0.415–1.216) | 0.213 | | | 0.633 (0.405–0.990) | 0.045 | 1.199 (0.552–2.607) | 0.646 |
| White blood cell ($10^3$/μL) | 1.103 (0.982–1.239) | 0.098 | | | 1.092 (0.977–1.220) | 0.121 | | |
| Platelet ($10^3$/μL) | 1.002 (0.998–1.006) | 0.278 | | | 1.001 (0.997–1.005) | 0.662 | | |
| $IgG_1$-G0F ≥40% (Yes = 1. No = 0) | 2.129 (1.093–4.150) | 0.026 | 0.855 (0.355–2.061) | 0.727 | 1.903 (1.078–3.359) | 0.026 | 0.746 (0.274–2.032) | 0.567 |
| $IgG_1$-G1F ≥30% (Yes = 1. No = 0) | 0.496 (0.258–0.955) | 0.036 | 1.181 (0.488–2.860) | 0.712 | 0.494 (0.275–0.888) | 0.018 | 1.606 (0.596–4.328) | 0.349 |
| $IgG_2$-G2FS >0% (Yes = 1. No = 0) | 1.713 (0.844–3.476) | 0.136 | | | 2.142 (1.128–4.070) | 0.020 | 1.302 (0.425–3.985) | 0.644 |

Abbreviations: CI, confidence interval; F, fucosylated; G0, agalactosylated; G1, partially galactosylated; G2, fully galactosylated; S, sialylated.

**Table S7.** Membrane protein identification in cholangiocarcinoma cell lines by mass spectrometry.

| Accession | Description | Score | Mass | Matches |
|---|---|---|---|---|
| *HuCCT1 cells* | | | | |
| 1C04 | HLA class I histocompatibility antigen, Cw-4 alpha chain | 2537 | 41368 | 30 |
| 1C18 | HLA class I histocompatibility antigen, Cw-18 alpha chain | 2462 | 41363 | 30 |
| EPCAM | Epithelial cell adhesion molecule | 1696 | 35594 | 24 |
| AT1A1 | Sodium/potassium-transporting ATPase subunit alpha-1 | 1059 | 114135 | 21 |
| CD44 | CD44 antigen | 839 | 82001 | 14 |
| 1A01 | HLA class I histocompatibility antigen, A-1 alpha chain | 772 | 41105 | 10 |
| VDAC2 | Voltage-dependent anion-selective channel protein 2 | 751 | 32060 | 11 |
| FLNB | Filamin-B | 627 | 280157 | 16 |
| SHC1 | SHC-transforming protein 1 | 621 | 63296 | 11 |
| CAZA1 | F-actin-capping protein subunit alpha-1 | 553 | 33073 | 11 |
| RLA2 | 60S acidic ribosomal protein P2 | 551 | 11658 | 18 |
| LAMB3 | Laminin subunit beta-3 | 540 | 133366 | 8 |
| SYPL1 | Synaptophysin-like protein 1 | 533 | 28889 | 14 |
| PGRC1 | Membrane-associated progesterone receptor component 1 | 516 | 21772 | 8 |
| SRPRB | Signal recognition particle receptor subunit beta | 489 | 29912 | 7 |
| PAR1 | Proteinase-activated receptor 1 | 455 | 48436 | 8 |
| TSP1 | Thrombospondin-1 | 429 | 133291 | 11 |
| ACTG | Actin, cytoplasmic 2 | 361 | 42108 | 18 |
| PLXB2 | Plexin-B2 | 358 | 207734 | 9 |
| INF2 | Inverted formin-2 | 357 | 136851 | 8 |
| ROA1 | Heterogeneous nuclear ribonucleoprotein A1 | 349 | 38837 | 5 |
| XRCC6 | X-ray repair cross-complementing protein 6 | 341 | 70084 | 5 |
| SEPT9 | Septin-9 | 302 | 65646 | 7 |
| SO4A1 | Solute carrier organic anion transporter family member 4A1 | 300 | 78796 | 6 |
| GP107 | Protein GPR107 | 282 | 67290 | 6 |
| CRTP1 | Cysteine-rich tail protein 1 | 279 | 16272 | 5 |
| MARCS | Myristoylated alanine-rich C-kinase substrate | 265 | 31707 | 3 |
| ANXA5 | Annexin A5 | 256 | 35971 | 7 |
| FLNA | Filamin-A | 254 | 283301 | 5 |
| LAT1 | Large neutral amino acids transporter small subunit 1 | 253 | 55659 | 5 |
| 4F2 | 4F2 cell-surface antigen heavy chain | 244 | 68180 | 5 |
| PANX1 | Pannexin-1 | 240 | 48646 | 5 |
| HSP7C | Heat shock cognate 71 kDa protein | 239 | 71082 | 10 |
| DSG2 | Desmoglein-2 | 235 | 123016 | 6 |
| PTRF | Polymerase I and transcript release factor | 234 | 43450 | 4 |
| NDRG3 | Protein NDRG3 | 218 | 41896 | 2 |

| | | | | |
|---|---|---|---|---|
| CKAP4 | Cytoskeleton-associated protein 4 | 216 | 66097 | 2 |
| TNR3 | Tumor necrosis factor receptor superfamily member 3 | 213 | 48104 | 3 |
| LMNA | Prelamin-A/C | 209 | 74380 | 4 |
| LAMA3 | Laminin subunit alpha-3 | 206 | 375652 | 5 |
| GDIA | Rab GDP dissociation inhibitor alpha | 205 | 51177 | 5 |
| ICAM1 | Intercellular adhesion molecule 1 | 200 | 58587 | 8 |
| 1433Z | 14-3-3 protein zeta/delta | 199 | 27899 | 16 |
| K2C8 | Keratin, type II cytoskeletal 8 | 194 | 53671 | 2 |
| GNAS1 | Guanine nucleotide-binding protein G(s) subunit alpha isoforms XLas | 194 | 111697 | 5 |
| DCBD2 | Discoidin, CUB and LCCL domain-containing protein 2 | 182 | 85893 | 2 |
| ITB1 | Integrin beta-1 | 181 | 91664 | 5 |
| SNP23 | Synaptosomal-associated protein 23 | 173 | 23682 | 4 |
| CTND1 | Catenin delta-1 | 169 | 108674 | 4 |
| SQSTM | Sequestosome-1 | 167 | 48455 | 3 |
| EGFR | Epidermal growth factor receptor | 166 | 137612 | 4 |
| DREB | Drebrin | 161 | 71842 | 2 |
| ANXA2 | Annexin A2 | 153 | 38808 | 3 |
| HYOU1 | Hypoxia up-regulated protein 1 | 152 | 111494 | 4 |
| JAM1 | Junctional adhesion molecule A | 151 | 32962 | 4 |
| PODXL | Podocalyxin | 142 | 59055 | 4 |
| ETFA | Electron transfer flavoprotein subunit alpha, mitochondrial | 132 | 35400 | 5 |
| MARK2 | Serine/threonine-protein kinase MARK2 | 131 | 88255 | 2 |
| ADAM9 | Disintegrin and metalloproteinase domain-containing protein 9 | 129 | 93006 | 5 |
| PRKDC | DNA-dependent protein kinase catalytic subunit | 119 | 473749 | 8 |
| 1433B | 14-3-3 protein beta/alpha | 118 | 28179 | 13 |
| LAP2 | Protein LAP2 | 116 | 158941 | 3 |
| DESP | Desmoplakin | 115 | 334021 | 5 |
| RFTN1 | Raftlin | 111 | 63677 | 1 |
| AT2C1 | Calcium-transporting ATPase type 2C member 1 | 98 | 101653 | 4 |
| 1B15 | HLA class I histocompatibility antigen, B-15 alpha chain | 96 | 40648 | 2 |
| TERA | Transitional endoplasmic reticulum ATPase | 96 | 89950 | 2 |
| ITA3 | Integrin alpha-3 | 94 | 117735 | 2 |
| LAMC1 | Laminin subunit gamma-1 | 93 | 183191 | 4 |
| VPP1 | V-type proton ATPase 116 kDa subunit a isoform 1 | 91 | 97148 | 4 |
| HS71A | Heat shock 70 kDa protein 1A | 87 | 70294 | 2 |
| MUC1 | Mucin-1 | 86 | 122200 | 9 |
| RHOG | Rho-related GTP-binding protein RhoG | 85 | 21751 | 6 |
| MYO1E | Unconventional myosin-Ie | 84 | 127552 | 2 |
| HNRPK | Heterogeneous nuclear ribonucleoprotein K | 79 | 51230 | 2 |

| | | | | |
|---|---|---|---|---|
| TFR1 | Transferrin receptor protein 1 | 77 | 85274 | 3 |
| DC1I2 | Cytoplasmic dynein 1 intermediate chain 2 | 76 | 71811 | 1 |
| PDIA1 | Protein disulfide-isomerase | 75 | 57480 | 3 |
| MOT4 | Monocarboxylate transporter 4 | 74 | 50064 | 3 |
| MBOA7 | Lysophospholipid acyltransferase 7 | 73 | 53415 | 2 |
| SPIT2 | Kunitz-type protease inhibitor 2 | 72 | 28951 | 3 |
| G3P | Glyceraldehyde-3-phosphate dehydrogenase | 71 | 36201 | 1 |
| PAXI | Paxillin | 70 | 65946 | 1 |
| KTN1 | Kinectin | 69 | 156464 | 1 |
| TACD2 | Tumor-associated calcium signal transducer 2 | 69 | 36371 | 1 |
| 1433G | 14-3-3 protein gamma | 69 | 28456 | 9 |
| A4 | Amyloid beta A4 protein | 68 | 87914 | 2 |
| PRDX6 | Peroxiredoxin-6 | 68 | 25133 | 3 |
| CD166 | CD166 antigen | 68 | 65745 | 2 |
| AIFM1 | Apoptosis-inducing factor 1, mitochondrial | 67 | 67144 | 3 |
| AHNK2 | Protein AHNAK2 | 63 | 617383 | 4 |
| GCN1L | Translational activator GCN1 | 62 | 294967 | 3 |
| MICB | MHC class I polypeptide-related sequence B | 58 | 43132 | 6 |
| LDHA | L-lactate dehydrogenase A chain | 54 | 36950 | 2 |
| T106B | Transmembrane protein 106B | 54 | 31393 | 1 |
| MYH9 | Myosin-9 | 53 | 227646 | 4 |
| CNPY2 | Protein canopy homolog 2 | 51 | 20981 | 1 |
| PAR2 | Proteinase-activated receptor 2 | 51 | 44611 | 4 |
| GRP78 | 78 kDa glucose-regulated protein | 51 | 72402 | 3 |
| TM9S4 | Transmembrane 9 superfamily member 4 | 49 | 75211 | 2 |
| NPC2 | Epididymal secretory protein E1 | 47 | 16902 | 5 |
| JIP4 | C-Jun-amino-terminal kinase-interacting protein 4 | 47 | 146913 | 1 |
| TOM34 | Mitochondrial import receptor subunit TOM34 | 47 | 34937 | 1 |
| RGS20 | Regulator of G-protein signaling 20 | 46 | 44291 | 1 |
| GCP60 | Golgi resident protein GCP60 | 42 | 60841 | 1 |
| SF3A1 | Splicing factor 3A subunit 1 | 42 | 88888 | 1 |
| DYL2 | Dynein light chain 2, cytoplasmic | 41 | 10457 | 1 |
| RRBP1 | Ribosome-binding protein 1 | 41 | 152780 | 1 |
| RAB5C | Ras-related protein Rab-5C | 40 | 23696 | 1 |
| HUWE1 | E3 ubiquitin-protein ligase HUWE1 | 40 | 485523 | 4 |
| RL29 | 60S ribosomal protein L29 | 39 | 17798 | 1 |
| VATH | V-type proton ATPase subunit H | 38 | 56417 | 2 |
| 1433S | 14-3-3 protein sigma | 37 | 27871 | 6 |
| ITA2 | Integrin alpha-2 | 36 | 130468 | 1 |

| | | | | |
|---|---|---|---|---|
| CH60 | 60 kDa heat shock protein, mitochondrial | 34 | 61187 | 1 |
| MMP1 | Interstitial collagenase | 33 | 54144 | 1 |
| IQGA1 | Ras GTPase-activating-like protein IQGAP1 | 33 | 189761 | 1 |
| KCNK1 | Potassium channel subfamily K member 1 | 30 | 38632 | 1 |
| EPS8 | Epidermal growth factor receptor kinase substrate 8 | 29 | 92167 | 1 |
| RHOA | Transforming protein RhoA | 29 | 22096 | 1 |
| RS27A | Ubiquitin-40S ribosomal protein S27a | 28 | 18296 | 1 |
| CXAR | Coxsackievirus and adenovirus receptor | 27 | 40575 | 1 |
| G6PI | Glucose-6-phosphate isomerase | 26 | 63335 | 1 |
| ENPL | Endoplasmin | 24 | 92696 | 1 |
| ACSL4 | Long-chain-fatty-acid--CoA ligase 4 | 24 | 80220 | 1 |
| CDCP1 | CUB domain-containing protein 1 | 22 | 94298 | 9 |
| IMB1 | Importin subunit beta-1 | 21 | 98420 | 2 |
| P2RX4 | P2X purinoceptor 4 | 21 | 44139 | 3 |
| TBA1A | Tubulin alpha-1A chain | 21 | 50788 | 3 |
| TENS4 | Tensin-4 | 19 | 77798 | 1 |
| PDLI5 | PDZ and LIM domain protein 5 | 18 | 65102 | 3 |
| CAPR2 | Caprin-2 | 16 | 126474 | 1 |
| PCBP1 | Poly(rC)-binding protein 1 | 15 | 37987 | 1 |
| AFAD | Afadin | 14 | 207702 | 1 |
| C2D1A | Coiled-coil and C2 domain-containing protein 1A | 13 | 104397 | 1 |

| **Accession** | **Description** | **Score** | **Mass** | **Matches** |
|---|---|---|---|---|
| *MMNK-1 cells* | | | | |
| AHNK | Neuroblast differentiation-associated protein AHNAK | 6290 | 629213 | 378 |
| ITB1 | Integrin beta-1 | 3291 | 91664 | 140 |
| ANXA2 | Annexin A2 | 3208 | 38808 | 120 |
| MYH9 | Myosin-9 | 2458 | 227646 | 179 |
| AT1A1 | Sodium/potassium-transporting ATPase subunit alpha-1 | 2188 | 114135 | 103 |
| TFR1 | Transferrin receptor protein 1 | 2087 | 85274 | 59 |
| AT1A2 | Sodium/potassium-transporting ATPase subunit alpha-2 | 2022 | 113505 | 70 |
| FLNA | Filamin-A | 1901 | 283301 | 150 |
| G3P | Glyceraldehyde-3-phosphate dehydrogenase | 1746 | 36201 | 112 |
| ACTB | Actin, cytoplasmic 1 | 1705 | 42052 | 87 |
| GBG12 | Guanine nucleotide-binding protein G(I)/G(S)/G(O) subunit gamma-12 | 1551 | 8115 | 38 |
| CD44 | CD44 antigen | 1549 | 82001 | 47 |
| 4F2 | 4F2 cell-surface antigen heavy chain | 1447 | 68180 | 35 |
| ACTC | Actin, alpha cardiac muscle 1 | 1245 | 42334 | 63 |
| TBB5 | Tubulin beta chain | 1149 | 50095 | 75 |
| TBB4B | Tubulin beta-4B chain | 1055 | 50255 | 73 |

| | | | | |
|---|---|---|---|---|
| 1433T | 14-3-3 protein theta | 1053 | 28032 | 40 |
| LAT1 | Large neutral amino acids transporter small subunit 1 | 1021 | 55659 | 27 |
| 1B15 | HLA class I histocompatibility antigen, B-15 alpha chain | 942 | 40648 | 76 |
| RAB5C | Ras-related protein Rab-5C | 924 | 23696 | 39 |
| VIME | Vimentin | 910 | 53676 | 81 |
| ANXA1 | Annexin A1 | 874 | 38918 | 31 |
| LDHA | L-lactate dehydrogenase A chain | 858 | 36950 | 67 |
| 1B48 | HLA class I histocompatibility antigen, B-48 alpha chain | 840 | 40679 | 43 |
| 1B57 | HLA class I histocompatibility antigen, B-57 alpha chain | 796 | 40541 | 62 |
| 1B58 | HLA class I histocompatibility antigen, B-58 alpha chain | 796 | 40597 | 63 |
| GNAS2 | Guanine nucleotide-binding protein G(s) subunit alpha isoforms short | 789 | 46092 | 40 |
| 1C12 | HLA class I histocompatibility antigen, Cw-12 alpha chain | 773 | 41316 | 62 |
| 1C17 | HLA class I histocompatibility antigen, Cw-17 alpha chain | 772 | 41612 | 58 |
| 1B27 | HLA class I histocompatibility antigen, B-27 alpha chain | 765 | 40802 | 60 |
| 1B73 | HLA class I histocompatibility antigen, B-73 alpha chain | 740 | 40809 | 53 |
| FLNC | Filamin-C | 708 | 293407 | 43 |
| MARCS | Myristoylated alanine-rich C-kinase substrate | 676 | 31707 | 27 |
| PDIA1 | Protein disulfide-isomerase | 668 | 57480 | 20 |
| NEP | Neprilysin | 632 | 86144 | 51 |
| TPIS | Triosephosphate isomerase | 618 | 31057 | 31 |
| MUC18 | Cell surface glycoprotein MUC18 | 610 | 72532 | 32 |
| HSP7C | Heat shock cognate 71 kDa protein | 601 | 71082 | 41 |
| CD151 | CD151 antigen | 592 | 29132 | 62 |
| TBB8 | Tubulin beta-8 chain | 592 | 50257 | 40 |
| TBB8L | Tubulin beta-8 chain-like protein LOC260334 | 592 | 50168 | 39 |
| BASP1 | Brain acid soluble protein 1 | 586 | 22680 | 51 |
| MOT4 | Monocarboxylate transporter 4 | 564 | 50064 | 46 |
| S10AB | Protein S100-A11 | 560 | 11847 | 42 |
| GRP78 | 78 kDa glucose-regulated protein | 553 | 72402 | 35 |
| MYH11 | Myosin-11 | 536 | 228054 | 81 |
| CTNB1 | Catenin beta-1 | 528 | 86069 | 41 |
| RRAS2 | Ras-related protein R-Ras2 | 519 | 23613 | 26 |
| TSN3 | Tetraspanin-3 | 509 | 28797 | 22 |
| HS71A | Heat shock 70 kDa protein 1A | 509 | 70294 | 26 |
| EF1A1 | Elongation factor 1-alpha 1 | 496 | 50451 | 40 |
| HS90A | Heat shock protein HSP 90-alpha | 484 | 85006 | 63 |
| MYO1C | Unconventional myosin-Ic | 468 | 122461 | 62 |
| 1433Z | 14-3-3 protein zeta/delta | 445 | 27899 | 21 |
| HS71L | Heat shock 70 kDa protein 1-like | 443 | 70730 | 23 |

| | | | | |
|---|---|---|---|---|
| HSP76 | Heat shock 70 kDa protein 6 | 443 | 71440 | 18 |
| 1A25 | HLA class I histocompatibility antigen, A-25 alpha chain | 436 | 41477 | 52 |
| 1B44 | HLA class I histocompatibility antigen, B-44 alpha chain | 425 | 40798 | 60 |
| 1B45 | HLA class I histocompatibility antigen, B-45 alpha chain | 425 | 40674 | 60 |
| YBOX1 | Nuclease-sensitive element-binding protein 1 | 422 | 35903 | 23 |
| IFM1 | Interferon-induced transmembrane protein 1 | 412 | 14126 | 16 |
| MYH10 | Myosin-10 | 406 | 229827 | 56 |
| FLNB | Filamin-B | 405 | 280157 | 50 |
| 5NTD | 5~-nucleotidase | 393 | 63898 | 42 |
| ROA1 | Heterogeneous nuclear ribonucleoprotein A1 | 387 | 38837 | 18 |
| STOM | Erythrocyte band 7 integral membrane protein | 377 | 31882 | 22 |
| GNAI2 | Guanine nucleotide-binding protein G(i) subunit alpha-2 | 366 | 40995 | 23 |
| POTEE | POTE ankyrin domain family member E | 348 | 122882 | 11 |
| 1433G | 14-3-3 protein gamma | 323 | 28456 | 24 |
| CALD1 | Caldesmon | 321 | 93232 | 25 |
| MYH14 | Myosin-14 | 319 | 228701 | 44 |
| MBOA7 | Lysophospholipid acyltransferase 7 | 317 | 53415 | 23 |
| ACTBL | Beta-actin-like protein 2 | 301 | 42318 | 11 |
| 1433B | 14-3-3 protein beta/alpha | 300 | 28179 | 29 |
| TF | Tissue factor | 298 | 33332 | 33 |
| ICAM1 | Intercellular adhesion molecule 1 | 298 | 58587 | 39 |
| LAMP1 | Lysosome-associated membrane glycoprotein 1 | 288 | 45367 | 20 |
| ALDOA | Fructose-bisphosphate aldolase A | 287 | 39851 | 16 |
| BASI | Basigin | 286 | 42573 | 17 |
| COR1C | Coronin-1C | 284 | 53899 | 39 |
| CDC42 | Cell division control protein 42 homolog | 273 | 21587 | 22 |
| GRP75 | Stress-70 protein, mitochondrial | 272 | 73920 | 38 |
| SEPT9 | Septin-9 | 272 | 65646 | 26 |
| LDHB | L-lactate dehydrogenase B chain | 268 | 36900 | 28 |
| 1433E | 14-3-3 protein epsilon | 268 | 29326 | 10 |
| NHRF1 | Na(+)/H(+) exchange regulatory cofactor NHE-RF1 | 264 | 39130 | 16 |
| SYPL1 | Synaptophysin-like protein 1 | 263 | 28889 | 21 |
| MAP4 | Microtubule-associated protein 4 | 263 | 121443 | 32 |
| 1433F | 14-3-3 protein eta | 260 | 28372 | 19 |
| 1433S | 14-3-3 protein sigma | 260 | 27871 | 17 |
| SEP11 | Septin-11 | 255 | 49652 | 14 |
| RS14 | 40S ribosomal protein S14 | 254 | 16434 | 19 |
| ITA3 | Integrin alpha-3 | 253 | 117735 | 41 |
| CTNA1 | Catenin alpha-1 | 253 | 100693 | 28 |

| | | | | |
|---|---|---|---|---|
| TAGL | Transgelin | 249 | 22653 | 28 |
| PODXL | Podocalyxin | 245 | 59055 | 37 |
| EZRI | Ezrin | 243 | 69484 | 30 |
| SPTB2 | Spectrin beta chain, non-erythrocytic 1 | 236 | 275237 | 44 |
| K2C8 | Keratin, type II cytoskeletal 8 | 234 | 53671 | 19 |
| AT2B4 | Plasma membrane calcium-transporting ATPase 4 | 233 | 139030 | 43 |
| TPM2 | Tropomyosin beta chain | 232 | 32945 | 25 |
| HS90B | Heat shock protein HSP 90-beta | 232 | 83554 | 21 |
| AT2B2 | Plasma membrane calcium-transporting ATPase 2 | 227 | 137987 | 34 |
| AT2B3 | Plasma membrane calcium-transporting ATPase 3 | 227 | 135253 | 36 |
| RAB35 | Ras-related protein Rab-35 | 221 | 23296 | 24 |
| E41L2 | Band 4.1-like protein 2 | 220 | 113032 | 49 |
| CKAP4 | Cytoskeleton-associated protein 4 | 216 | 66097 | 29 |
| GBB1 | Guanine nucleotide-binding protein G(I)/G(S)/G(T) subunit beta-1 | 214 | 38151 | 32 |
| CTNA2 | Catenin alpha-2 | 212 | 106045 | 16 |
| TPM4 | Tropomyosin alpha-4 chain | 209 | 28619 | 33 |
| MYL6 | Myosin light polypeptide 6 | 208 | 17090 | 15 |
| H90B4 | Putative heat shock protein HSP 90-beta 4 | 207 | 58855 | 10 |
| DREB | Drebrin | 205 | 71842 | 14 |
| ATPA | ATP synthase subunit alpha, mitochondrial | 204 | 59828 | 24 |
| ANLN | Actin-binding protein anillin | 203 | 125490 | 49 |
| SC22B | Vesicle-trafficking protein SEC22b | 201 | 24806 | 28 |
| RAB32 | Ras-related protein Rab-32 | 201 | 25210 | 27 |
| GLU2B | Glucosidase 2 subunit beta | 200 | 60357 | 20 |
| CH60 | 60 kDa heat shock protein, mitochondrial | 194 | 61187 | 18 |
| K2C75 | Keratin, type II cytoskeletal 75 | 194 | 59809 | 15 |
| PHB2 | Prohibitin-2 | 193 | 33276 | 24 |
| RL13 | 60S ribosomal protein L13 | 183 | 24304 | 9 |
| FLOT2 | Flotillin-2 | 182 | 47434 | 21 |
| L1CAM | Neural cell adhesion molecule L1 | 179 | 140885 | 28 |
| TPM3 | Tropomyosin alpha-3 chain | 179 | 32987 | 21 |
| MRP | MARCKS-related protein | 179 | 19574 | 9 |
| ZN532 | Zinc finger protein 532 | 177 | 144001 | 21 |
| RL18A | 60S ribosomal protein L18a | 176 | 21034 | 11 |
| MYCB2 | E3 ubiquitin-protein ligase MYCBP2 | 173 | 517856 | 15 |
| ANX11 | Annexin A11 | 172 | 54697 | 22 |
| CTND1 | Catenin delta-1 | 168 | 108674 | 28 |
| SQSTM | Sequestosome-1 | 165 | 48455 | 23 |
| UTRO | Utrophin | 163 | 396444 | 30 |

| | | | | |
|---|---|---|---|---|
| MYOF | Myoferlin | 159 | 236100 | 23 |
| AAAT | Neutral amino acid transporter B(0) | 159 | 57018 | 14 |
| SCRB2 | Lysosome membrane protein 2 | 152 | 54712 | 14 |
| CALR | Calreticulin | 149 | 48283 | 17 |
| VDAC2 | Voltage-dependent anion-selective channel protein 2 | 143 | 32060 | 11 |
| S43A3 | Solute carrier family 43 member 3 | 142 | 55063 | 9 |
| GNAI3 | Guanine nucleotide-binding protein G(k) subunit alpha | 141 | 41076 | 19 |
| VATL | V-type proton ATPase 16 kDa proteolipid subunit | 141 | 15725 | 9 |
| CD63 | CD63 antigen | 140 | 26474 | 31 |
| RS27A | Ubiquitin-40S ribosomal protein S27a | 135 | 18296 | 25 |
| MOES | Moesin | 135 | 67892 | 33 |
| PDIA3 | Protein disulfide-isomerase A3 | 133 | 57146 | 21 |
| RAC1 | Ras-related C3 botulinum toxin substrate 1 | 129 | 21835 | 7 |
| SPTN1 | Spectrin alpha chain, non-erythrocytic 1 | 126 | 285163 | 49 |
| CLIC4 | Chloride intracellular channel protein 4 | 125 | 28982 | 8 |
| ACSL4 | Long-chain-fatty-acid--CoA ligase 4 | 124 | 80220 | 25 |
| BPTF | Nucleosome-remodeling factor subunit BPTF | 124 | 340791 | 13 |
| CFA99 | Cilia- and flagella-associated protein 99 | 124 | 52489 | 10 |
| RAB5B | Ras-related protein Rab-5B | 124 | 23920 | 8 |
| RB11A | Ras-related protein Rab-11A | 123 | 24492 | 16 |
| CALM | Calmodulin | 122 | 16827 | 9 |
| CLH1 | Clathrin heavy chain 1 | 122 | 193260 | 16 |
| EPHA2 | Ephrin type-A receptor 2 | 119 | 109679 | 11 |
| RAP1A | Ras-related protein Rap-1A | 119 | 21316 | 18 |
| LEG1 | Galectin-1 | 118 | 15048 | 17 |
| TLN1 | Talin-1 | 117 | 271766 | 35 |
| H2B1C | Histone H2B type 1-C/E/F/G/I | 116 | 13898 | 23 |
| NPM | Nucleophosmin | 115 | 32726 | 18 |
| RHOG | Rho-related GTP-binding protein RhoG | 114 | 21751 | 9 |
| CHD1 | Chromodomain-helicase-DNA-binding protein 1 | 110 | 197707 | 13 |
| TBA1A | Tubulin alpha-1A chain | 110 | 50788 | 40 |
| GBB2 | Guanine nucleotide-binding protein G(I)/G(S)/G(T) subunit beta-2 | 109 | 38048 | 14 |
| IF4A1 | Eukaryotic initiation factor 4A-I | 109 | 46353 | 10 |
| SC61B | Protein transport protein Sec61 subunit beta | 109 | 10025 | 12 |
| ARF4 | ADP-ribosylation factor 4 | 106 | 20612 | 6 |
| PHB | Prohibitin | 105 | 29843 | 5 |
| RL29 | 60S ribosomal protein L29 | 103 | 17798 | 8 |
| ACTN1 | Alpha-actinin-1 | 103 | 103563 | 20 |
| NPTN | Neuroplastin | 103 | 44702 | 15 |

| | | | | |
|---|---|---|---|---|
| ACTN4 | Alpha-actinin-4 | 102 | 105245 | 15 |
| LIMA1 | LIM domain and actin-binding protein 1 | 102 | 85630 | 24 |
| IQGA1 | Ras GTPase-activating-like protein IQGAP1 | 98 | 189761 | 15 |
| HLAH | Putative HLA class I histocompatibility antigen, alpha chain H | 97 | 41094 | 38 |
| NAV2 | Neuron navigator 2 | 96 | 269314 | 39 |
| MAML2 | Mastermind-like protein 2 | 96 | 125404 | 39 |
| HNRPK | Heterogeneous nuclear ribonucleoprotein K | 95 | 51230 | 10 |
| ADT2 | ADP/ATP translocase 2 | 94 | 33059 | 30 |
| PROF1 | Profilin-1 | 93 | 15216 | 5 |
| FLOT1 | Flotillin-1 | 91 | 47554 | 15 |
| MT1E | Metallothionein-1E | 91 | 7150 | 9 |
| MT1G | Metallothionein-1G | 91 | 7277 | 9 |
| TCPQ | T-complex protein 1 subunit theta | 89 | 60153 | 8 |
| POTEI | POTE ankyrin domain family member I | 88 | 122858 | 6 |
| QCR1 | Cytochrome b-c1 complex subunit 1, mitochondrial | 84 | 53297 | 8 |
| AT134 | Probable cation-transporting ATPase 13A4 | 83 | 135725 | 21 |
| TPM1 | Tropomyosin alpha-1 chain | 82 | 32746 | 12 |
| H4 | Histone H4 | 81 | 11360 | 4 |
| DHCR7 | 7-dehydrocholesterol reductase | 81 | 55195 | 4 |
| CNN3 | Calponin-3 | 81 | 36562 | 17 |
| GBB4 | Guanine nucleotide-binding protein subunit beta-4 | 80 | 38284 | 5 |
| ENOA | Alpha-enolase | 79 | 47481 | 10 |
| SC11A_ | Signal peptidase complex catalytic subunit SEC11A | 78 | 20612 | 2 |
| RRBP1 | Ribosome-binding protein 1 | 78 | 152780 | 24 |
| LMNA_ | Prelamin-A/C | 77 | 74380 | 18 |
| CAD13 | Cadherin-13 | 77 | 78694 | 9 |
| MPRI | Cation-independent mannose-6-phosphate receptor | 77 | 281155 | 7 |
| NUCL | Nucleolin | 77 | 76625 | 6 |
| COF1 | Cofilin-1 | 76 | 18719 | 11 |
| RAB1A | Ras-related protein Rab-1A | 74 | 22891 | 4 |
| EXTL1 | Exostosin-like 1 | 74 | 75391 | 9 |
| KPYM | Pyruvate kinase PKM | 73 | 58470 | 18 |
| RS28 | 40S ribosomal protein S28 | 72 | 7893 | 9 |
| H2A1A | Histone H2A type 1-A | 71 | 14225 | 16 |
| RS19 | 40S ribosomal protein S19 | 69 | 16051 | 14 |
| SERPH | Serpin H1 | 69 | 46525 | 7 |
| PASD1 | PAS domain-containing protein 1 | 68 | 88286 | 5 |
| CC175 | Coiled-coil domain-containing protein 175 | 68 | 94023 | 5 |
| PLCX2 | PI-PLC X domain-containing protein 2 | 68 | 35154 | 4 |

| | | | | |
|---|---|---|---|---|
| FKBP3 | Peptidyl-prolyl cis-trans isomerase FKBP3 | 68 | 25218 | 7 |
| CLDN1 | Claudin domain-containing protein 1 | 68 | 29268 | 2 |
| STML2 | Stomatin-like protein 2, mitochondrial | 67 | 38624 | 10 |
| ENOB | Beta-enolase | 67 | 47299 | 5 |
| LASP1 | LIM and SH3 domain protein 1 | 66 | 30097 | 9 |
| DCR1C | Protein artemis | 66 | 79413 | 8 |
| RAB8B | Ras-related protein Rab-8B | 65 | 23740 | 2 |
| DC1I2 | Cytoplasmic dynein 1 intermediate chain 2 | 65 | 71811 | 4 |
| PGK1 | Phosphoglycerate kinase 1 | 64 | 44985 | 24 |
| SRPRB | Signal recognition particle receptor subunit beta | 64 | 29912 | 1 |
| ANXA6 | Annexin A6 | 64 | 76168 | 28 |
| A4 | Amyloid beta A4 protein | 63 | 87914 | 3 |
| ENPL | Endoplasmin | 62 | 92696 | 10 |
| RL8 | 60S ribosomal protein L8 | 62 | 28235 | 1 |
| LPPRC | Leucine-rich PPR motif-containing protein, mitochondrial | 60 | 159003 | 4 |
| CP4Z1 | Cytochrome P450 4Z1 | 60 | 59618 | 16 |
| UBE2O | E2/E3 hybrid ubiquitin-protein ligase UBE2O | 60 | 142631 | 4 |
| RFTN1 | Raftlin | 59 | 63677 | 22 |
| CD47 | Leukocyte surface antigen CD47 | 58 | 35590 | 12 |
| PLAK | Junction plakoglobin | 56 | 82434 | 7 |
| ITB3 | Integrin beta-3 | 56 | 90194 | 6 |
| MYL1 | Myosin light chain 1/3, skeletal muscle isoform | 56 | 21189 | 3 |
| RAB8A | Ras-related protein Rab-8A | 56 | 23824 | 20 |
| VAMP2 | Vesicle-associated membrane protein 2 | 56 | 12712 | 5 |
| GFAP | Glial fibrillary acidic protein | 55 | 49907 | 7 |
| CAZA1 | F-actin-capping protein subunit alpha-1 | 55 | 33073 | 15 |
| PDIA4 | Protein disulfide-isomerase A4 | 54 | 73229 | 10 |
| NACA2 | Nascent polypeptide-associated complex subunit alpha-2 | 54 | 23209 | 4 |
| NACAM | Nascent polypeptide-associated complex subunit alpha, muscle-specific form | 54 | 205979 | 4 |
| ITA6 | Integrin alpha-6 | 54 | 127724 | 18 |
| LAMP2 | Lysosome-associated membrane glycoprotein 2 | 54 | 45503 | 13 |
| RAB14 | Ras-related protein Rab-14 | 53 | 24110 | 6 |
| SCRIB | Protein scribble homolog | 53 | 175748 | 8 |
| TM109 | Transmembrane protein 109 | 53 | 26194 | 14 |
| QCR2 | Cytochrome b-c1 complex subunit 2, mitochondrial | 53 | 48584 | 5 |
| CD99 | CD99 antigen | 52 | 18893 | 2 |
| CH10 | 10 kDa heat shock protein, mitochondrial | 50 | 10925 | 7 |
| ANXA4 | Annexin A4 | 50 | 36088 | 4 |
| ATPG | ATP synthase subunit gamma, mitochondrial | 50 | 33032 | 4 |

| | | | | |
|---|---|---|---|---|
| HMGA1 | High mobility group protein HMG-I/HMG-Y | 49 | 11669 | 18 |
| TM245 | Transmembrane protein 245 | 49 | 101508 | 2 |
| SYNEM | Synemin | 49 | 173005 | 19 |
| AT12A | Potassium-transporting ATPase alpha chain 2 | 49 | 116292 | 8 |
| DYST | Dystonin | 49 | 865259 | 31 |
| DDX3Y | ATP-dependent RNA helicase DDX3Y | 49 | 73564 | 7 |
| DDX3X | ATP-dependent RNA helicase DDX3X | 49 | 73597 | 6 |
| LMO7 | LIM domain only protein 7 | 49 | 194002 | 12 |
| OSTM1 | Osteopetrosis-associated transmembrane protein 1 | 48 | 38031 | 6 |
| GBG7 | Guanine nucleotide-binding protein G(I)/G(S)/G(O) subunit gamma-7 | 47 | 7631 | 2 |
| RAB1B | Ras-related protein Rab-1B | 47 | 22328 | 5 |
| VDAC1 | Voltage-dependent anion-selective channel protein 1 | 47 | 30868 | 11 |
| RL13A | 60S ribosomal protein L13a | 47 | 23619 | 1 |
| HIF3A | Hypoxia-inducible factor 3-alpha | 46 | 73072 | 10 |
| EGFR | Epidermal growth factor receptor | 46 | 137612 | 19 |
| EF2 | Elongation factor 2 | 46 | 96246 | 14 |
| NONO | Non-POU domain-containing octamer-binding protein | 45 | 54311 | 11 |
| 2AAA | Serine/threonine-protein phosphatase 2A 65 kDa regulatory subunit A alpha isoform | 45 | 66065 | 10 |
| ARF5 | ADP-ribosylation factor 5 | 45 | 20631 | 2 |
| ARF1 | ADP-ribosylation factor 1 | 45 | 20741 | 2 |
| LRRF1 | Leucine-rich repeat flightless-interacting protein 1 | 45 | 89826 | 6 |
| ATPB | ATP synthase subunit beta, mitochondrial | 44 | 56525 | 5 |
| PLP2 | Proteolipid protein 2 | 44 | 17022 | 7 |
| PTRF | Polymerase I and transcript release factor | 44 | 43450 | 16 |
| TBB6 | Tubulin beta-6 chain | 44 | 50281 | 9 |
| HCD2 | 3-hydroxyacyl-CoA dehydrogenase type-2 | 44 | 27134 | 5 |
| EIF3B | Eukaryotic translation initiation factor 3 subunit B | 43 | 92823 | 2 |
| ZYX | Zyxin | 43 | 62436 | 15 |
| DYH8 | Dynein heavy chain 8, axonemal | 43 | 517984 | 26 |
| DYH5 | Dynein heavy chain 5, axonemal | 43 | 532504 | 10 |
| DCA13 | DDB1- and CUL4-associated factor 13 | 43 | 51996 | 5 |
| RC3H1 | Roquin-1 | 43 | 126627 | 2 |
| ACTZ | Alpha-centractin | 43 | 42701 | 4 |
| FA53B | Protein FAM53B | 43 | 46993 | 2 |
| PTBP1 | Polypyrimidine tract-binding protein 1 | 43 | 57357 | 8 |
| RALA | Ras-related protein Ral-A | 43 | 23723 | 12 |
| SSBP | Single-stranded DNA-binding protein, mitochondrial | 43 | 17249 | 6 |
| EF1D | Elongation factor 1-delta | 43 | 31217 | 6 |

| | | | | |
|---|---|---|---|---|
| HSPB1 | Heat shock protein beta-1 | 43 | 22826 | 2 |
| SHRM3 | Protein Shroom3 | 42 | 218321 | 16 |
| TPD54 | Tumor protein D54 | 42 | 22281 | 20 |
| TMED2 | Transmembrane emp24 domain-containing protein 2 | 42 | 22860 | 4 |
| DDAH1 | N(G),N(G)-dimethylarginine dimethylaminohydrolase 1 | 42 | 31444 | 3 |
| H33 | Histone H3.3 | 42 | 15376 | 2 |
| RL12 | 60S ribosomal protein L12 | 42 | 17979 | 4 |
| PGAM1 | Phosphoglycerate mutase 1 | 42 | 28900 | 11 |
| STK10 | Serine/threonine-protein kinase 10 | 42 | 112749 | 4 |
| CERKL | Ceramide kinase-like protein | 42 | 63324 | 7 |
| LTOR2 | Ragulator complex protein LAMTOR2 | 41 | 13613 | 7 |
| CNN1 | Calponin-1 | 41 | 33321 | 6 |
| PAIRB | Plasminogen activator inhibitor 1 RNA-binding protein | 41 | 44995 | 5 |
| CS018 | Uncharacterized protein C19orf18 | 40 | 24307 | 8 |
| H31T | Histone H3.1t | 40 | 15613 | 11 |
| RAB5A | Ras-related protein Rab-5A | 40 | 23872 | 11 |
| H90B2 | Putative heat shock protein HSP 90-beta 2 | 40 | 44492 | 3 |
| HIP1R | Huntingtin-interacting protein 1-related protein | 40 | 119999 | 19 |
| RYR1 | Ryanodine receptor 1 | 40 | 570517 | 13 |
| MYH15 | Myosin-15 | 40 | 225904 | 16 |
| INCE | Inner centromere protein | 40 | 105764 | 15 |
| AFAD | Afadin | 40 | 207702 | 12 |
| TTBK1 | Tau-tubulin kinase 1 | 40 | 142992 | 13 |
| SH24B | SH2 domain-containing protein 4B | 40 | 51314 | 8 |
| KRT84 | Keratin, type II cuticular Hb4 | 40 | 65942 | 8 |
| SMUF1 | E3 ubiquitin-protein ligase SMURF1 | 40 | 86915 | 8 |
| THAP3 | THAP domain-containing protein 3 | 40 | 27384 | 6 |
| CO6A2 | Collagen alpha-2(VI) chain | 40 | 109709 | 7 |
| MA7D1 | MAP7 domain-containing protein 1 | 40 | 93106 | 8 |
| WWC2 | Protein WWC2 | 40 | 134835 | 19 |
| NOP14 | Nucleolar protein 14 | 40 | 98292 | 7 |
| DDRGK | DDRGK domain-containing protein 1 | 40 | 35589 | 6 |
| RHG35 | Rho GTPase-activating protein 35 | 40 | 171946 | 7 |
| CODA1 | Collagen alpha-1(XIII) chain | 40 | 70363 | 4 |
| DCTN2 | Dynactin subunit 2 | 40 | 44318 | 3 |
| YBOX3 | Y-box-binding protein 3 | 40 | 40066 | 5 |
| YBOX2 | Y-box-binding protein 2 | 40 | 38552 | 5 |
| PLEC | Plectin | 39 | 533462 | 63 |
| IMDH2 | Inosine-5~-monophosphate dehydrogenase 2 | 39 | 56226 | 9 |

| | | | | |
|---|---|---|---|---|
| PRIO | Major prion protein | 39 | 27871 | 1 |
| RHOA | Transforming protein RhoA | 39 | 22096 | 3 |
| RL3 | 60S ribosomal protein L3 | 39 | 46365 | 9 |
| RS3A | 40S ribosomal protein S3a | 38 | 30154 | 15 |
| RRAS | Ras-related protein R-Ras | 38 | 23637 | 4 |
| STT3A | Dolichyl-diphosphooligosaccharide--protein glycosyltransferase subunit STT3A | 38 | 81104 | 10 |
| ARP3B | Actin-related protein 3B | 37 | 48090 | 10 |
| RL28 | 60S ribosomal protein L28 | 37 | 15795 | 8 |
| H13 | Histone H1.3 | 37 | 22336 | 7 |
| MYO1B | Unconventional myosin-Ib | 37 | 132928 | 9 |
| DSG2 | Desmoglein-2 | 37 | 123016 | 8 |
| H14 | Histone H1.4 | 37 | 21852 | 12 |
| H12 | Histone H1.2 | 37 | 21352 | 8 |
| M3K1 | Mitogen-activated protein kinase kinase kinase 1 | 37 | 166419 | 3 |
| RLA2 | 60S acidic ribosomal protein P2 | 37 | 11658 | 15 |
| H1T | Histone H1t | 37 | 22006 | 3 |
| RAVR1 | Ribonucleoprotein PTB-binding 1 | 36 | 64464 | 1 |
| RL27 | 60S ribosomal protein L27 | 36 | 15788 | 1 |
| BCAM | Basal cell adhesion molecule | 36 | 68161 | 3 |
| TCPD | T-complex protein 1 subunit delta | 36 | 58401 | 16 |
| CTR1 | High affinity cationic amino acid transporter 1 | 36 | 68449 | 1 |
| ERP29 | Endoplasmic reticulum resident protein 29 | 36 | 29032 | 11 |
| TRI38 | E3 ubiquitin-protein ligase TRIM38 | 36 | 54636 | 8 |
| AKP13 | A-kinase anchor protein 13 | 36 | 310381 | 33 |
| GNA12 | Guanine nucleotide-binding protein subunit alpha-12 | 36 | 44422 | 4 |
| GNAL | Guanine nucleotide-binding protein G(olf) subunit alpha | 36 | 44794 | 3 |
| GNA13 | Guanine nucleotide-binding protein subunit alpha-13 | 36 | 44364 | 3 |
| HNRPM | Heterogeneous nuclear ribonucleoprotein M | 35 | 77749 | 9 |
| LEG3 | Galectin-3 | 35 | 26193 | 4 |
| ESYT1 | Extended synaptotagmin-1 | 35 | 123293 | 3 |
| MIF | Macrophage migration inhibitory factor | 35 | 12639 | 1 |
| TCPG | T-complex protein 1 subunit gamma | 35 | 61066 | 11 |
| SEPT2 | Septin-2 | 35 | 41689 | 13 |
| NUDC | Nuclear migration protein nudC | 35 | 38276 | 2 |
| STXB1 | Syntaxin-binding protein 1 | 35 | 67925 | 3 |
| ARHG2 | Rho guanine nucleotide exchange factor 2 | 35 | 112386 | 12 |
| VINC | Vinculin | 34 | 124292 | 15 |
| QSOX2 | Sulfhydryl oxidase 2 | 34 | 78221 | 6 |
| HPCA | Neuron-specific calcium-binding protein hippocalcin | 34 | 22527 | 5 |

| | | | | |
|---|---|---|---|---|
| FETUA | Alpha-2-HS-glycoprotein | 34 | 40098 | 2 |
| CKAP5 | Cytoskeleton-associated protein 5 | 34 | 227062 | 11 |
| ECHM | Enoyl-CoA hydratase, mitochondrial | 33 | 31823 | 2 |
| S38AA | Putative sodium-coupled neutral amino acid transporter 10 | 33 | 120371 | 13 |
| REEP5 | Receptor expression-enhancing protein 5 | 33 | 21707 | 4 |
| K1C18 | Keratin, type I cytoskeletal 18 | 33 | 48029 | 20 |
| ROA2 | Heterogeneous nuclear ribonucleoproteins A2/B1 | 33 | 37464 | 3 |
| SEPT7 | Septin-7 | 33 | 50933 | 17 |
| MCAF1 | Activating transcription factor 7-interacting protein 1 | 33 | 137166 | 4 |
| NPC1 | Niemann-Pick C1 protein | 33 | 144868 | 6 |
| DCBD2 | Discoidin, CUB and LCCL domain-containing protein 2 | 33 | 85893 | 1 |
| AT5G1 | ATP synthase F(0) complex subunit C1, mitochondrial | 32 | 14439 | 2 |
| RS21 | 40S ribosomal protein S21 | 32 | 9220 | 4 |
| PVRL2 | Nectin-2 | 32 | 58162 | 2 |
| SIN3B | Paired amphipathic helix protein Sin3b | 32 | 134067 | 8 |
| RL4 | 60S ribosomal protein L4 | 32 | 47953 | 11 |
| B2MG | Beta-2-microglobulin | 32 | 13820 | 4 |
| PGRC1 | Membrane-associated progesterone receptor component 1 | 32 | 21772 | 3 |
| KI16B | Kinesin-like protein KIF16B | 32 | 152488 | 14 |
| CFA43 | Cilia- and flagella-associated protein 43 | 32 | 193686 | 28 |
| CP2CJ | Cytochrome P450 2C19 | 32 | 56636 | 20 |
| NALCN | Sodium leak channel non-selective protein | 32 | 202196 | 12 |
| PDLI7 | PDZ and LIM domain protein 7 | 32 | 50896 | 3 |
| CSRP1 | Cysteine and glycine-rich protein 1 | 32 | 21409 | 2 |
| STMN1 | Stathmin | 31 | 17292 | 5 |
| STMN2 | Stathmin-2 | 31 | 20929 | 4 |
| TMEM2 | Transmembrane protein 2 | 31 | 155702 | 12 |
| ERD21 | ER lumen protein-retaining receptor 1 | 31 | 24754 | 1 |
| TM7S3 | Transmembrane 7 superfamily member 3 | 31 | 64752 | 2 |
| NFIP2 | NEDD4 family-interacting protein 2 | 31 | 36709 | 7 |
| TTC9B | Tetratricopeptide repeat protein 9B | 31 | 26201 | 4 |
| CE164 | Centrosomal protein of 164 kDa | 30 | 164727 | 22 |
| CYC | Cytochrome c | 30 | 11855 | 10 |
| SRC8 | Src substrate cortactin | 30 | 61720 | 9 |
| PLCB3 | 1-phosphatidylinositol 4,5-bisphosphate phosphodiesterase beta-3 | 30 | 139511 | 9 |
| RN214 | RING finger protein 214 | 30 | 78360 | 5 |
| ZW10 | Centromere/kinetochore protein zw10 homolog | 30 | 89628 | 6 |
| RUFY2 | RUN and FYVE domain-containing protein 2 | 30 | 75806 | 8 |
| MRCKG | Serine/threonine-protein kinase MRCK gamma | 30 | 173778 | 6 |

| | | | | |
|---|---|---|---|---|
| KBL | 2-amino-3-ketobutyrate coenzyme A ligase, mitochondrial | 30 | 45826 | 5 |
| RL32 | 60S ribosomal protein L32 | 30 | 15964 | 2 |
| HKDC1 | Putative hexokinase HKDC1 | 30 | 103790 | 6 |
| EXOC1 | Exocyst complex component 1 | 30 | 102772 | 11 |
| AKAP2 | A-kinase anchor protein 2 | 30 | 95002 | 9 |
| KINH | Kinesin-1 heavy chain | 30 | 110358 | 4 |
| JKIP3 | Janus kinase and microtubule-interacting protein 3 | 30 | 98640 | 5 |
| TAGL3_ | Transgelin-3 | 30 | 22629 | 11 |
| PA2G4 | Proliferation-associated protein 2G4 | 30 | 44101 | 2 |
| ANXA5 | Annexin A5 | 30 | 35971 | 2 |
| RPN2 | Dolichyl-diphosphooligosaccharide--protein glycosyltransferase subunit 2 | 30 | 69355 | 4 |
| KTN1 | Kinectin | 30 | 156464 | 16 |
| CYR61 | Protein CYR61 | 30 | 44165 | 3 |
| DMRT1 | Doublesex- and mab-3-related transcription factor 1 | 29 | 40018 | 2 |
| ACACA | Acetyl-CoA carboxylase 1 | 29 | 267095 | 17 |
| TLK2 | Serine/threonine-protein kinase tousled-like 2 | 29 | 88405 | 14 |
| RRP5 | Protein RRP5 homolog | 29 | 209939 | 21 |
| ASPP2 | Apoptosis-stimulating of p53 protein 2 | 29 | 126222 | 16 |
| VILI | Villin-1 | 29 | 93093 | 4 |
| DAF | Complement decay-accelerating factor | 29 | 42400 | 4 |
| RL34 | 60S ribosomal protein L34 | 29 | 13513 | 25 |
| FSBP | Fibrinogen silencer-binding protein | 29 | 34860 | 5 |
| SYCP2 | Synaptonemal complex protein 2 | 28 | 177239 | 13 |
| ZN844 | Zinc finger protein 844 | 28 | 78708 | 7 |
| PPIA | Peptidyl-prolyl cis-trans isomerase A | 28 | 18229 | 7 |
| CDCP1 | CUB domain-containing protein 1 | 28 | 94298 | 5 |
| NHRF2 | Na(+)/H(+) exchange regulatory cofactor NHE-RF2 | 28 | 37619 | 1 |
| BD1L1 | Biorientation of chromosomes in cell division protein 1-like 1 | 28 | 332433 | 10 |
| NIN | Ninein | 28 | 245208 | 5 |
| PCNT | Pericentrin | 28 | 380600 | 6 |
| LIMC1 | LIM and calponin homology domains-containing protein 1 | 28 | 122818 | 4 |
| EMP3 | Epithelial membrane protein 3 | 28 | 18816 | 2 |
| RL30 | 60S ribosomal protein L30 | 28 | 12947 | 3 |
| SYSM | Serine--tRNA ligase, mitochondrial | 28 | 58702 | 7 |
| OFD1 | Oral-facial-digital syndrome 1 protein | 28 | 117055 | 5 |
| WDR60 | WD repeat-containing protein 60 | 28 | 123294 | 3 |
| TRAF4 | TNF receptor-associated factor 4 | 28 | 55218 | 6 |
| CD59 | CD59 glycoprotein | 28 | 14795 | 3 |
| RS4X | 40S ribosomal protein S4, X isoform | 28 | 29807 | 5 |

| | | | | |
|---|---|---|---|---|
| RS4Y1 | 40S ribosomal protein S4, Y isoform 1 | 28 | 29665 | 10 |
| RL26L | 60S ribosomal protein L26-like 1 | 28 | 17246 | 5 |
| DCD | Dermcidin | 28 | 11391 | 5 |
| VAMP5 | Vesicle-associated membrane protein 5 | 27 | 12968 | 8 |
| CYTSB | Cytospin-B | 27 | 119197 | 7 |
| GANAB | Neutral alpha-glucosidase AB | 27 | 107263 | 2 |
| TRXR1 | Thioredoxin reductase 1, cytoplasmic | 27 | 71832 | 32 |
| OR2W1 | Olfactory receptor 2W1 | 27 | 36704 | 29 |
| RAB7A | Ras-related protein Rab-7a | 27 | 23760 | 6 |
| RL3L | 60S ribosomal protein L3-like | 27 | 46609 | 9 |
| NCF4 | Neutrophil cytosol factor 4 | 27 | 39121 | 16 |
| TAGL2 | Transgelin-2 | 27 | 22548 | 9 |
| RADI | Radixin | 27 | 68635 | 11 |
| EXOSX | Exosome component 10 | 27 | 101566 | 27 |
| TLDC1 | TLD domain-containing protein 1 | 27 | 51588 | 1 |
| CUX2 | Homeobox protein cut-like 2 | 27 | 162091 | 13 |
| ZNT1 | Zinc transporter 1 | 27 | 56290 | 9 |
| ERG7 | Lanosterol synthase | 26 | 84453 | 3 |
| DDX5 | Probable ATP-dependent RNA helicase DDX5 | 26 | 69618 | 5 |
| AT1B3 | Sodium/potassium-transporting ATPase subunit beta-3 | 26 | 31834 | 29 |
| RS3 | 40S ribosomal protein S3 | 26 | 26842 | 3 |
| 2AAB | Serine/threonine-protein phosphatase 2A 65 kDa regulatory subunit A beta isoform | 26 | 66799 | 12 |
| PDE9A | High affinity cGMP-specific 3~,5~-cyclic phosphodiesterase 9A | 26 | 69475 | 4 |
| BAZ1B | Tyrosine-protein kinase BAZ1B | 26 | 172392 | 13 |
| HNRPC | Heterogeneous nuclear ribonucleoproteins C1/C2 | 26 | 33707 | 11 |
| CTRL | Chymotrypsin-like protease CTRL-1 | 25 | 28554 | 3 |
| SH3L3 | SH3 domain-binding glutamic acid-rich-like protein 3 | 25 | 10488 | 7 |
| PAXI | Paxillin | 25 | 65946 | 1 |
| SYNCI | Syncoilin | 25 | 55494 | 10 |
| CRBG3 | Very large A-kinase anchor protein | 25 | 333107 | 21 |
| CTL2 | Choline transporter-like protein 2 | 25 | 81610 | 3 |
| RS9 | 40S ribosomal protein S9 | 25 | 22635 | 1 |
| TNPO1 | Transportin-1 | 25 | 103771 | 13 |
| RPN1 | Dolichyl-diphosphooligosaccharide--protein glycosyltransferase subunit 1 | 24 | 68641 | 9 |
| OBSCN | Obscurin | 24 | 879630 | 18 |
| RTEL1 | Regulator of telomere elongation helicase 1 | 24 | 134968 | 10 |
| T183B | Transmembrane protein 183B | 24 | 43539 | 4 |
| FERM2 | Fermitin family homolog 2 | 24 | 78438 | 8 |

| | | | | |
|---|---|---|---|---|
| NOMO1 | Nodal modulator 1 | 24 | 135209 | 11 |
| TGON2 | Trans-Golgi network integral membrane protein 2 | 24 | 51082 | 5 |
| HNRPQ | Heterogeneous nuclear ribonucleoprotein Q | 24 | 69788 | 2 |
| SRSF3 | Serine/arginine-rich splicing factor 3 | 24 | 19546 | 3 |
| FAS | Fatty acid synthase | 24 | 275877 | 2 |
| MACF1 | Microtubule-actin cross-linking factor 1, isoforms 1/2/3/5 | 24 | 843033 | 57 |
| FINC | Fibronectin | 24 | 266052 | 9 |
| ZBED2 | Zinc finger BED domain-containing protein 2 | 24 | 25278 | 11 |
| QCR6 | Cytochrome b-c1 complex subunit 6, mitochondrial | 24 | 11017 | 1 |
| IF122 | Intraflagellar transport protein 122 homolog | 24 | 143787 | 8 |
| CD166 | CD166 antigen | 24 | 65745 | 9 |
| RL21 | 60S ribosomal protein L21 | 24 | 18610 | 3 |
| ZO2 | Tight junction protein ZO-2 | 23 | 134104 | 2 |
| NLRC4 | NLR family CARD domain-containing protein 4 | 23 | 117396 | 24 |
| AP2B1 | AP-2 complex subunit beta | 23 | 105398 | 18 |
| AP1B1 | AP-1 complex subunit beta-1 | 23 | 105482 | 18 |
| CD9 | CD9 antigen | 23 | 25969 | 3 |
| AGRIN | Agrin | 23 | 225246 | 16 |
| CHAP1 | Chromosome alignment-maintaining phosphoprotein 1 | 23 | 90012 | 10 |
| RASH | GTPase HRas | 23 | 21627 | 4 |
| ICE1 | Little elongation complex subunit 1 | 23 | 250759 | 18 |
| CAP1 | Adenylyl cyclase-associated protein 1 | 23 | 52325 | 9 |
| CLPT1 | Cleft lip and palate transmembrane protein 1 | 22 | 76277 | 1 |
| ARF6 | ADP-ribosylation factor 6 | 22 | 20183 | 1 |
| RS5 | 40S ribosomal protein S5 | 22 | 23033 | 4 |
| SND1 | Staphylococcal nuclease domain-containing protein 1 | 22 | 102618 | 3 |
| MTMR5 | Myotubularin-related protein 5 | 22 | 210294 | 2 |
| S39AE | Zinc transporter ZIP14 | 22 | 54918 | 3 |
| VASP | Vasodilator-stimulated phosphoprotein | 22 | 39976 | 5 |
| DUS26 | Dual specificity protein phosphatase 26 | 21 | 24101 | 3 |
| AHNK2 | Protein AHNAK2 | 21 | 617383 | 17 |
| EXOC4 | Exocyst complex component 4 | 21 | 111170 | 1 |
| H15 | Histone H1.5 | 21 | 22566 | 6 |
| K1C19 | Keratin, type I cytoskeletal 19 | 21 | 44079 | 1 |
| TERA | Transitional endoplasmic reticulum ATPase | 21 | 89950 | 22 |
| ZN185 | Zinc finger protein 185 | 21 | 74393 | 8 |
| PCDA6 | Protocadherin alpha-6 | 21 | 103336 | 6 |
| CS047 | Uncharacterized protein C19orf47 | 21 | 44890 | 1 |
| RBM34 | RNA-binding protein 34 | 21 | 48649 | 11 |

| | | | | |
|---|---|---|---|---|
| NIBAN | Protein Niban | 21 | 104039 | 10 |
| PYR1 | CAD protein | 20 | 245167 | 1 |
| FDFT | Squalene synthase | 20 | 48597 | 1 |
| UBR5 | E3 ubiquitin-protein ligase UBR5 | 20 | 312352 | 25 |
| CD14 | Monocyte differentiation antigen CD14 | 20 | 40678 | 3 |
| TX264 | Testis-expressed sequence 264 protein | 20 | 34452 | 1 |
| CYFP1 | Cytoplasmic FMR1-interacting protein 1 | 19 | 146742 | 2 |
| CCD66 | Coiled-coil domain-containing protein 66 | 19 | 110256 | 19 |
| UBXN4 | UBX domain-containing protein 4 | 19 | 57028 | 3 |
| VAT1 | Synaptic vesicle membrane protein VAT-1 homolog | 18 | 42122 | 3 |
| FA49B | Protein FAM49B | 18 | 37010 | 6 |
| ZN208 | Zinc finger protein 208 | 18 | 152123 | 11 |
| PRS38 | Serine protease 38 | 18 | 35960 | 1 |
| OXSR1 | Serine/threonine-protein kinase OSR1 | 18 | 58271 | 3 |
| IF2B3 | Insulin-like growth factor 2 mRNA-binding protein 3 | 18 | 64008 | 8 |
| TMEDA | Transmembrane emp24 domain-containing protein 10 | 18 | 25131 | 1 |
| GNA11 | Guanine nucleotide-binding protein subunit alpha-11 | 18 | 42382 | 12 |
| GNA14 | Guanine nucleotide-binding protein subunit alpha-14 | 18 | 42000 | 15 |
| R3HCL | Coiled-coil domain-containing protein R3HCC1L | 18 | 88855 | 11 |
| CMTR1 | Cap-specific mRNA (nucleoside-2~-O-)-methyltransferase 1 | 17 | 96172 | 29 |
| PTMA | Prothymosin alpha | 17 | 12196 | 2 |
| TXND5 | Thioredoxin domain-containing protein 5 | 17 | 48283 | 2 |
| TKT | Transketolase | 16 | 68519 | 1 |
| KRT35 | Keratin, type I cuticular Ha5 | 16 | 51640 | 2 |
| RT4I1 | Reticulon-4-interacting protein 1, mitochondrial | 16 | 43961 | 7 |
| GI24 | Platelet receptor Gi24 | 16 | 34343 | 4 |
| CXAR | Coxsackievirus and adenovirus receptor | 16 | 40575 | 2 |
| SYEP | Bifunctional glutamate/proline--tRNA ligase | 16 | 172080 | 12 |
| HYOU1 | Hypoxia up-regulated protein 1 | 16 | 111494 | 8 |
| RL7A | 60S ribosomal protein L7a | 16 | 30148 | 3 |
| STXB2 | Syntaxin-binding protein 2 | 16 | 66867 | 4 |
| PTK7 | Inactive tyrosine-protein kinase 7 | 16 | 119799 | 2 |
| VIGLN | Vigilin | 15 | 141995 | 2 |
| GPC1 | Glypican-1 | 15 | 62724 | 2 |
| SYMC | Methionine--tRNA ligase, cytoplasmic | 15 | 102249 | 1 |
| IF2B1 | Insulin-like growth factor 2 mRNA-binding protein 1 | 15 | 63783 | 9 |
| RAB13 | Ras-related protein Rab-13 | 15 | 22988 | 3 |
| PRKDC | DNA-dependent protein kinase catalytic subunit | 15 | 473749 | 28 |
| S39AA | Zinc transporter ZIP10 | 15 | 94928 | 3 |

| | | | | |
|---|---|---|---|---|
| HMCN1 | Hemicentin-1 | 14 | 623265 | 46 |
| EPHB4 | Ephrin type-B receptor 4 | 14 | 109741 | 5 |
| TITIN | Titin | 14 | 3842904 | 104 |
| SC23A | Protein transport protein Sec23A | 14 | 87018 | 1 |
| HECD1 | E3 ubiquitin-protein ligase HECTD1 | 14 | 292225 | 26 |
| SFXN2 | Sideroflexin-2 | 14 | 36493 | 8 |
| APOL5 | Apolipoprotein L5 | 14 | 47413 | 1 |
| MOT1 | Monocarboxylate transporter 1 | 13 | 54593 | 4 |
| OST48 | Dolichyl-diphosphooligosaccharide--protein glycosyltransferase 48 kDa subunit | 13 | 50940 | 8 |
| ZP1 | Zona pellucida sperm-binding protein 1 | 13 | 71202 | 2 |
| CA2D3 | Voltage-dependent calcium channel subunit alpha-2/delta-3 | 13 | 124074 | 3 |

**Table S8.** Levels of tumor-associated cytokines in healthy controls (n = 55) and patients with cholangiocarcinoma (n = 60).

| Cytokine (pg/mL) | Healthy Control | Cholangiocarcinoma | *p*-Value |
|---|---|---|---|
| IL-4 | 0.0 (0.0–47.9) | 0.0 (0.0–93.3) | <0.001 |
| IL-6 | 0.0 (0.0–92.8) | 1.6 (0.0–129.8) | 0.046 |
| IL-10 | 0.0 (0.0–93.4) | 0.0 (0.0–350.7) | 0.047 |
| IFN-$\gamma$ | 0.0 (0.0–343.4) | 212.9 (0.0–1216.7) | <0.001 |
| TGF-$\beta$1 | 12.3 (0.0–481.7) | 89.5 (0.0–750.0) | 0.045 |
| TNF-$\alpha$ | 0.0 (0.0–382.1) | 70.8 (25.3–220.7) | <0.001 |

Data are median values (minimum–maximum). Variables are compared using Mann-Whitney *U* tests. Abbreviations: IFN, interferon; IL, interleukin; TGF, transforming growth factor; TNF, tumor necrosis factor.

© 2018 by the authors. Licensee MDPI, Basel, Switzerland. This article is an open access article distributed under the terms and conditions of the Creative Commons Attribution (CC BY) license (http://creativecommons.org/licenses/by/4.0/).
